# Global Role of Vanadium for Cryptogamic Nitrogen Fixation in Extratropical Forests

**DOI:** 10.1021/acs.est.5c12982

**Published:** 2026-03-10

**Authors:** Romain Darnajoux, Shannon J. Haynes, Marie Renaudin, Nicolas Magain, Sessina Dani, Spencer Koonin, Jolanta Miadlikowska, Yoshitaka Uchida, Takamitsu Ohigashi, Diane Haughland, François Lutzoni, Jean-Philippe Bellenger, Xinning Zhang

**Affiliations:** 1 Department of Geosciences, 6740 Princeton University, Princeton, New Jersey 08544, United States; 2 High Meadows Environmental Institute, Princeton University, Princeton, New Jersey 08544, United States; 3 Centre Sève, Université de Sherbrooke, Sherbrooke, QC J1K 2R1, Canada; 4 Département de Chimie, Université de Sherbrooke, Sherbrooke, QC J1K 2R1, Canada; 5 InBioS Research Center, Université de Liège, Liège 4000, Belgium; 6 Department of Biology, 3065 Duke University, Durham, North Carolina 27708, United States; 7 Research Faculty School of Agriculture, 12810 Hokkaido University, Sapporo 060-0808, Japan; 8 Alberta Biodiversity Monitoring Institute, University of Alberta, Edmonton, AB T6G 2E9, Canada; 9 Department of Renewable Resources, University of Alberta, Edmonton, AB T6G 2H1, Canada

**Keywords:** molybdenum, vanadium, alternative nitrogenase, trace metal, temperature, cryptogamic covers, nonvascular phototrophs

## Abstract

Biological nitrogen fixation (BNF) by nitrogenase is often assumed to rely on molybdenum as an enzymatic cofactor, despite molybdenum scarcity in terrestrial ecosystems relative to vanadium and iron, two alternative cofactors. Findings that cyanolichens across northeastern American boreal forests can rely substantially on vanadium nitrogenase (V-Nase) for BNF suggest that V-Nase is used by other cryptogams, which collectively contribute a large share of terrestrial BNF. Here, we show global-scale vanadium-based nitrogen fixation in common cryptogams from deciduous and needleleaf extratropical forests, including remote and urban areas. Measurements demonstrate V-Nase activity in bryophytes and cyanolichens from 44 sites across three continents. V-Nase is regulated by molybdenum content and nitrogen fixation rate, a marker of nitrogen demand. Extrapolations based on nutrient deposition suggest hotspots for V-Nase activity at higher latitudes and nonsignificant contributions in urbanized areas (>40% and <10% sample BNF, respectively) and a likely decrease in sample-specific activities by 30% relative to preindustrial times. This newly discovered global role of vanadium forces a re-evaluation of the past, present, and future nitrogen cycle. Including nitrogenase enzymatic heterogeneity can help close the nitrogen budget, predict future forest productivity, and model the response of the terrestrial carbon sink to global change, particularly at high latitudes.

## Graphical abstract

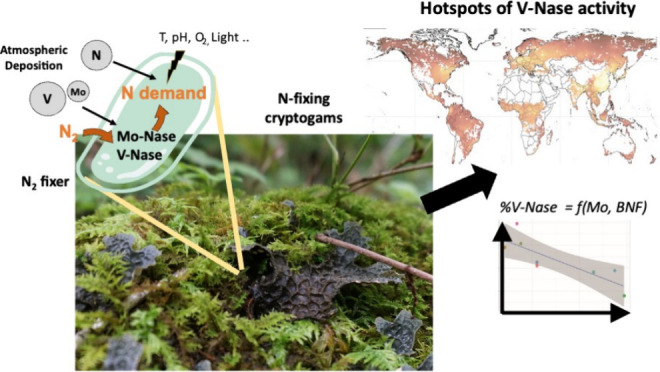

## Introduction

As a common limiting nutrient in extratropical ecosystems,^1,2^ nitrogen (N) greatly influences the magnitude of the carbon sink,^3,4^ particularly in rapidly changing mountainous and high latitude environments.^5^ Biological nitrogen fixation (BNF) by cryptogamic ground (e.g., biocrust) and vegetation covers (e.g., lichens and mosses) is a crucial source of new nitrogen; it is estimated to contribute between 15 and 50% of the total BNF in terrestrial ecosystems globally^6,7^ and up to 50% of total N input in high latitude environments.^8,9^ Yet, our understanding of cryptogamic BNF remains insufficient considering its broad contribution to critical ecosystem services, including carbon and nutrient sequestration, organic matter decomposition, and pathogen control.^10,11^

In cryptogamic species and free-living N fixers, efforts to quantify N input by BNF and understand its environmental controls often assume sole contribution from the molybdenum (Mo)-based nitrogenase.^12^ This is despite the genetic presence in various environments of two “alternative” enzyme isoforms,^13–16^ the vanadium (V) and iron (Fe)-only nitrogenases (Nases), that have different activities and regulatory mechanisms.^17^ Because of the different isozyme calibration factors (*R*_Mo_ = 3–4, *R*_V_= 1.5–2, *R*_Fe_ < 0.5) for the most common method to assess nitrogenase activity (acetylene reduction assay),^18^ disregarding nitrogenase diversity can lead to substantially underestimated N input and hinder identification of the factors that modulate N entry into ecosystems.^13^ It is thus crucial to investigate Mo-independent nitrogenase contributions to BNF and the biotic and abiotic factors regulating their activities in natural habitats, particularly in extratropical areas where moss- and lichen-associated BNF is important.

The study of these alternative nitrogenases in natural environmental samples was historically made difficult by the inability to quantify the activity of each isozyme separately, but modern isotopic methods, the isotopic acetylene reduction assay (ISARA) and its updated version low-activity-ISARA (LISARA), now allow the activity of Mo-dependent and Mo-independent nitrogenases to be accurately distinguished.^19,20^ Both methods are based on the different carbon isotopic fractionations of nitrogenase isozymes for acetylene reduction to ethylene (−1‰ vs −8‰ and −6‰ for the Mo, V, and Fe-Nase, respectively).^18^ Hence, the activity of alternative nitrogenase, documented in laboratory model organisms for over 30 years,^21^ has only recently been studied in the field and is increasingly recognized as a complementary route for N input to canonical Mo-based BNF.^15,19,22–24^ Isotopic methods do not yet distinguish V-and Fe-only Nases activities. Since Fe-Nase has never been found in cyanobacteria, the microorganisms typically responsible for BNF in cryptogams, it is likely that Mo-independent activity is due solely to the V-Nase enzyme.

The first regional scale study of V-Nase in cryptogamic organisms from the cyanolichen genus *Peltigera*demonstrated large, variable contributions to BNF for specimens collected across the northeastern American boreal biome (average 30%, range 0–80%).^22^ This study also validated the role of V-Nase in sustaining BNF under Mo limitation in natural environments and identified a Mo threshold value (∼250 μg_Mo_.g_Lichen_^–1^) below which V-Nase activity was likely to occur. As cryptogamic covers lack roots and must receive their micronutrients from atmospheric deposition and nearby vegetation, it is likely that similar threshold values exist across cryptogamic species, including bryophytes which are more abundant than cyanolichens and have exhibited V-Nase activity in preliminary research.^20^ *In vitro* and pure culture experiments have identified temperature as an additional factor that can affect the regulation and relative activity of Mo- and V-Nases, with temperatures below 15–20 °C favoring the latter.^25–27^ The role of temperature on the relative contributions of the different Nases to BNF has yet to be tested in cryptogamic species that have more complex trophic relationships than free-living N-fixers. This is particularly important in fast warming high-latitude areas where mosses and lichens play an important role in N biogeochemistry.^8,9,28^ Given that Mo is scarce^29^ and often limiting for BNF^30^ in terrestrial ecosystems (including in mosses and lichens),^31,32^ the annual mean temperature in boreal and temperate ecosystems is lower than 15 °C, and the frequent detection of V-Nase genes in terrestrial environments,^15^ we hypothesized that V-Nase is likely to be active and important in extratropical forests globally and that Mo and temperature are global drivers of this activity across diverse cryptogamic covers and forest habitats.

A first quantification of V-based BNF in extratropical cryptogam groups is attainable using LISARA^17^ and will improve our understanding of trace metal–nitrogen biogeochemical coupling. Coupling V-Nase contribution to BNF with global estimates of group-specific cryptogamic BNF rates will ultimately help constrain regional and global BNF input budgets (range 43–208 Tg N yr^–1^)^6,7^ and refine BNF representations in future Earth system models.

To evaluate the contribution of V-Nase to the N_2_ fixation of cryptogamic covers from extratropical forests, we applied the LISARA method to >700 specimens of lichens and bryophytes collected from over 40 sites across 9 locations located on three continents. Our sampling campaign focused on the most abundant species within these environments, spanned several latitudes and altitudinal gradients, representing a diversity of evergreen and deciduous forests subject to contrasting levels of atmospheric Mo deposition (Figure 1A).^33^ We specifically investigated the role of Mo in controlling sample V-Nase activity across our survey and tested the effect of incubation temperature (range 10–30 °C) on the short-term change in Nase isoform usage in boreal moss and lichen species. The results enabled us to propose a simple mechanistic framework to predict V-Nase contributions based on organism-scale Mo content and BNF rate and identify locations of potentially high V-Nase contribution to cryptogam BNF across extratropical forests globally, including during preindustrial times.

**Figure 1:**
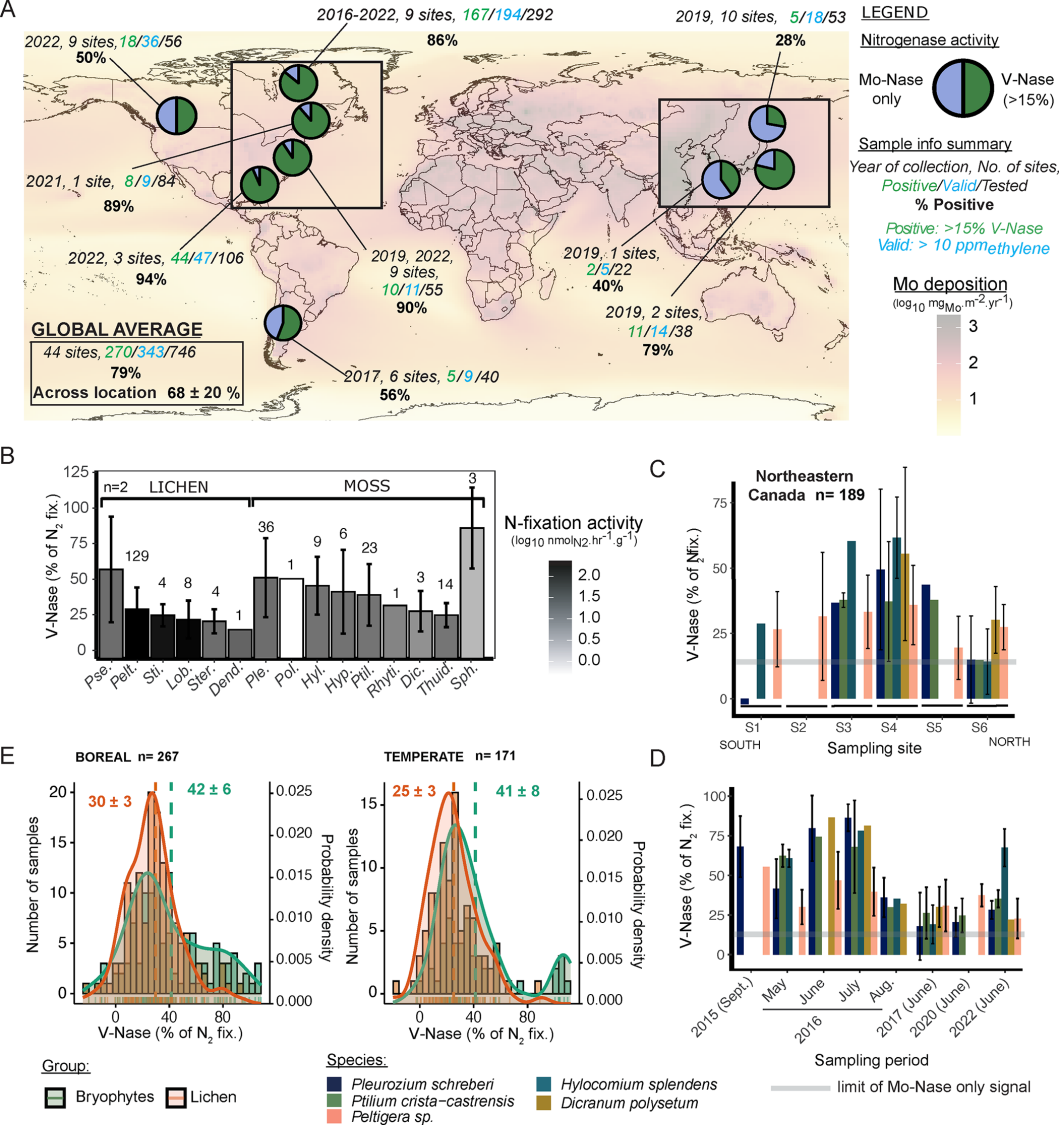
Contribution of vanadium Nase (V-Nase) to specimen BNF in cryptogamic covers of extratropical forests. (A) Geographic location of sampling sites within molybdenum deposition gradients and summary statistics of sample Nase activities (see Tables S1–S3). Bottom left inset indicates the average contribution across all locations with SD (*n* = 9). (B) V-Nase contributions to sample BNF per lichen and moss genus (*Pseudocyphellaria* (*Pse.*), *Peltigera* (*Pelt.*), *Lobaria* (*Lob.*), *Sticta* (*Sti.*), *Stereocaulon* (*Ster.*), *Dendriscosticta* (*Dend.*), *Politricum* (*Pol.*), *Hylocomium* (*Hyl.*), *Pleurozium* (*Ple.*), *Dicranum* (*Dic.*), *Hypnum* (*Hyp.*), *Ptilium* (*Ptil.*), *Rhytidiadelphus* (*Rhyti.*), *Thuidium* (*Thuid.*), and *Sphagnum* (*Sph.*)). (C, D) V-Nase contribution to BNF for samples from Northeastern Canada collected at (C) 6 sites along a 600 km transect (SI Figure S1) and (D) between 2016 and 2022 at site S4 (*n* = 184). Thick gray line represents the higher CI_95%_ for Mo-Nase only activity. (E) Ecosystem-specific distribution of V-Nase contributions (Average_Group_ ± CI_95%_). Error bars are one SD.

(A) adapted from the model of Wong et al.^33^

## Materials and Methods

### Description of Survey Campaigns and Sample Collection

From September 2015 to September 2022, samples of bryophytes (mosses, including *Sphagnum*, and liverworts) and cyanolichens (bimembered and trimembered, containing primarly cyanobacteria or green algae and cyanobacteria as their photobionts) were collected at nine forested locations in North America (Alberta, Québec, New Hampshire, New Jersey, and North Carolina), South America (Chile), and East Asia (Hokkaido, Honshu, and Kyushu in Japan) (see site information and sampling details in SI Supplementary Methods S1 and Tables S1–S4). Most locations were visited based on the known or suspected presence of *Peltigera* cyanolichens, a model for the study of V-Nase activity in the environment.^22^ Other locations with lower moss abundance and no cyanolichens in more anthropized areas were also visited. At seven of the nine locations, several sites separated by >10 km were visited, while at the other two locations, only one site was visited, for a total of 44 sites. In Québec, several sites were visited five times from 2015 to 2022 (details and site location in SI Figure S1 and Methods S1). Within each site, multiple subsites located more than 100 m apart were defined based on prioritizing moss abundance and cyanolichens, for a total of 109 sampling plots with an average of 5 samples per sampling plot (median = 4, min = 1, max = 125).

As only limited information is available in the literature on the contribution of moss and lichen species to nitrogen fixation in temperate areas, we focused our sampling on the most abundant species present at each site and specifically targeted a diversity of substrates (standing or fallen trees, rocks, or on forest floors) and canopy openness status to integrate the effects of small-scale heterogeneity on metal dynamics and BNF activity within each sampling plot.^34–36^ Specimens were collected under field conditions in a brown paper bag and allowed to air-dry until no evidence of moisture was observed. Sample bags were subsequently closed and stored in a dark and dry place until further processing in the laboratory (timing between collection and experiments was less than 4 weeks). In total, 830 samples were collected for the entirety of this study (509 bryophyte and 321 lichen samples), and 746 samples were used for the survey analyses (469 bryophyte and 277 lichen samples, Tables S2–S4, Figure 1).

Moss (*Pleurozium schreberi*, *Ptilium crista-castrensis*, *Hylocomium splendens*, *Dicranum polysetum*, *Polytrichum commune*, *Hypnum cupressiforme*, and *Climacium americanum*) and lichen (*Peltigera aphthosa*, *Lobaria pulmonaria*) samples with recognizable morphologies were identified at the species level. Other lichen (*Peltigera* spp., *Sticta* spp., *Leptogium* spp., *Lobaria* spp., *Nephroma* spp., *Pseudocyphellaria* spp*., Stereocolon* spp) and bryophyte (e.g., *Sphagnum* spp., *Dicranum* spp., *Conocephalum* spp., *Plagiomnium* spp., *Thuidium* spp., *Rhytidiadelphus* spp.) specimens were recognizable morphologically only at the genus or family level for the purpose of this study.

Several samples from eastern USA (New Jersey: *n* = 32, New Hampshire: *n* = 9) were included in the original paper of the LISARA method.^20^ Samples published by Darnajoux et al.^22^ were included in the statistical analyses (as found in Figure 3 and SI Table S7). All details about individual samples, including previously published data samples, can be found in Data Set S1.

### Acetylene Reduction Assay (ARA) Incubations

Within one month of each sampling campaign, between 50 and 150 collected samples were selected for enzyme activity assessments (0.08–0.3 g_DW_), integrating for species diversity and number of sites and subsites visited. Priority was given to cyanolichen and moss species with the reported ability to fix N (e.g., *Pleurozium* sp., *Ptilium* sp., and *Hypnum* sp.). Samples were processed for nitrogen fixation measurements (24 h) using the acetylene reduction assay (ARA),^37^ following the protocol described by Chen et al.^38^ with a few modifications. Briefly, samples were first rewetted to saturation, with excess water removed, and left to preincubate for 10–12 h at room temperature before the onset of the ARA. After 24 h of laboratory incubation with 10% v/v acetylene under constant light, headspace (15–18 mL) was removed from the vials and transferred first to a 12 mL brown glass serum vial (10 mL, Wheaton, Fisher Scientific) fitted with a 20 mm blue butyl septa (Bellco Glass) for ethylene δ^13^C isotopic analyses and then to a 5 mL Exetainer tube for ethylene concentration quantification. On the day of the incubation, several aliquots of the source acetylene were saved in a 12 mL brown glass vial or as a 10% v/v dilution in a 27 mL serum bottle, both fitted with blue butyl septa. These reference samples were kept in the dark until further processing. In 2016, all samples (only site S4 collected) were incubated under field conditions following the same gas sampling protocol described above, with a few modifications (see SI Methods S2 for details on all ARA incubation).

### Effect of Temperature on Nase Activity

To test the effect of temperature on the activities of the Mo- and V-Nases, dedicated samples of *Peltigera* sp. lichens (15 bags) and*Pleurozium schreberi*and*Ptilium crista-castrensis*mosses (20 bags each) were collected in large quantities at 20 plots across three sites in Quebec (S1, S4, and S6) in June 2020. Matching samples of similar mass (*m*_lichen_ = 0.18 ± 0.07 g, *m*_moss_ = 0.23 ± 0.04 g, average ± SD) were prepared from each bag and incubated at 10, 20, or 30 °C during the revival and ARA incubation periods. All samples were incubated in temperature-controlled incubators (Infors HT Multitron Pro) under constant light. Lichen samples were tested at an additional temperature of 15 °C. Each sample of moss and lichen was processed according to the methodology described above.

### Low Activity Isotopic Acetylene Reduction Assay Analyses

Mo- and Mo-independent Nase contributions to BNF activity were quantified following the ISARA method developed by Zhang et al.^19^ and later modified by Haynes et al. to improve its sensitivity (LISARA, limit of detection <10 ppm ethylene).^20^ Briefly, the method relies on the isotopic fractionation of ^13^C during the reduction of acetylene into ethylene (^13^ε_*AR*_), which is specific to each isozyme (^13^ε_*AR*, *Mo*_ = −14‰, ^13^ε_*AR*, *V*_= −8‰, and ^13^ε_*AR*, *Fe*_= −6‰), to estimate the contributions of Mo and non-Mo nitrogenase to the acetylene reduction activity recorded. Carbon isotopic compositions were measured on a homemade Gas Bench system interfaced to a Delta V GC-C-IRMS as described in Haynes et al.^20^ Samples yielding at least 10 ppmv ethylene could be analyzed by LISARA (referred to as the “Valid” sample, Tables S3–S4).

### Estimates of V-Nase Contribution to Sample BNF and BNF Rate Correction

Contributions of non-Mo nitrogenase to acetylene reduction and to N_2_ reduction were calculated according to Haynes et al.^20^ as summarized in SI Supplementary Methods S2. We assumed no Fe-Nase contribution in lichens and mosses^14–16,24^ and thus expressed all our results as the contribution of V-Nase toward N_2_ (%V-Nase_BNF_). The measured ^13^ε_*AR*_ for each sample was converted using isoform-specific values into the share of the total acetylene reduction activity (%V-Nase_AR_) (see SI Methods S2). Across the seven years, the samples were gathered, and improvement in the calibration methodology led to a slight difference in the calculation of %V-Nase_AR_ for different groups of samples (see Tables S3 and S4).

Nitrogen fixation activity corrected for V-Nase contribution was calculated using %V-Nase_AR_ and the ethylene reduction rate according to eq 1.

$${\mathrm{BNF}}_{N2}=\frac{{\mathrm{BNF}}_{\mathrm{AR}}\times \%{VNase}_{AR}}{R_{V}}+\frac{{\mathrm{BNF}}_{\mathrm{AR}}\times \left(1-\%{VNase}_{AR}\right)}{R_{Mo}}$$ 1

with *R*_Mo_ = 4 and *R*_V_ = 2 for the in vivo calibration factor between acetylene and dinitrogen reduction for Mo- and V-Nase, respectively.^18^ These values are consistent with those from mutant strains,^18^ bimodal distribution of *R* ratio in mosses,^39^ and ^15^N_2,g_ calibration conducted on cyanolichens.^13^

The contribution toward N fixation (%V-Nase_BNF_) was obtained by using eq 2.

%VNaseBNF=%VNaseARRV%VNaseARRV+(1−%VNaseAR)RMo 2

Total uncertainty of the % VNase_BNF_ is ∼15% for all the data obtained after 2019, and ∼20% for data from 2015 to 2017 (Figure 1C–D) (refer to Haynes et al., Supporting Information,^20^ for a full uncertainty assessment).

### Metal Content of Cryptogamics Covers

Following LISARA incubation, moss and lichen samples were freeze-dried and ground, and 20–40 mg of sample was digested using trace metal grade nitric acid (Thermo Fisher Scientific) and 10% Optima H_2_O_2_ (Thermo Fisher Scientific) in a 15 mL DigiTube (SCP Science) on a sand bath at 90 ± 10 °C until all organic material was dissolved (2–4 h, visual assessment). Digested samples (3 mL) were diluted to 20% v/v using Milli-Q water, and aliquots at 2% v/v (1 mL in 10 mL) were sent to the Laboratory for Isotopes and Metals in the Environment (LIME) at Penn State University (Pennsylvania, USA) for Mo analyses. Samples from Chile (*n* = 10) were sent as solid material and digested directly at the LIME following a similar protocol. A subset of samples from New Jersey (*n* = 32) were digested using a microwave assisted digestion system (MARS, CEM) and sent to the LIME. Peach leaves (NIST SRM 1547) and procedural blanks were used to assess the baseline, recovery, precision, and accuracy of the analyses. To limit the effect of contamination in our conclusion (see Supporting Discussion S1), we used the median and median absolute deviation as a robust estimator of centrality and dispersion for all Mo data.

### Assessment of V-Nase Gene Expression

To measure *vnf* gene expression by moss-associated cyanobacteria (Figure 2B), *Pleurozium schreberi* and *Ptilium crista-castrensis* moss samples were collected at two sites in the northeastern Canadian boreal forest in June and September 2017. Brown decaying moss shoot parts were discarded; green parts were placed in sterile cryogenic tubes and flash-frozen in liquid nitrogen on the site shortly after collection. Samples were subsequently processed following the protocol described in Renaudin et al.^40^ qPCR conditions are summarized in SI Supplementary Methods S3.

**Figure 2:**
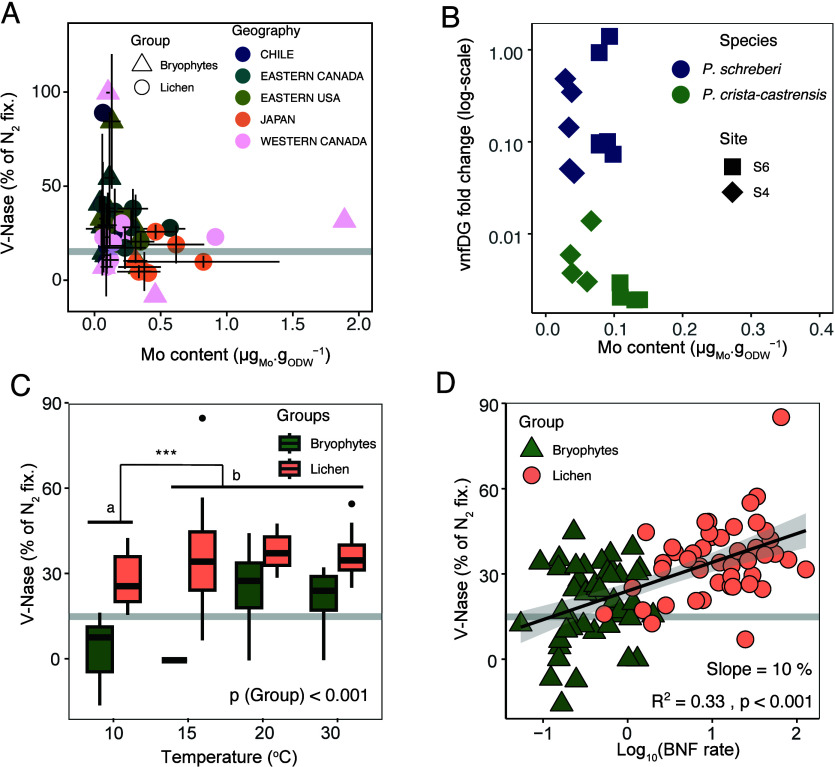
Environmental controls over specimen V-Nase activity in cryptogams from extratropical forests. (A) Role of molybdenum content (median and MAD) on V-Nase contribution (mean + SD) to specimen BNF across sites (*n* = 40). (B) V-Nase gene (*vnf*DG) expression in boreal moss species, *Pleurozium schreberi* and *Ptilium crista-castrensis*, at 2 sites in Northeastern Canada (*n* = 18). (C) Temperature effects on V-Nase contributions to sample BNF in *P. schreberi* and *P. crista-castrensis* mosses and *Peltigera* spp. cyanolichens (*n* = 91). (D) Effect of sample BNF rate (log_10_ nmol_N2_ h^–1^ g_sample_^–1^) on V-Nase contribution (*n* = 91). In (A), (C), and (D), the thick gray line indicates higher CI_95%_ for Mo-Nase only activity. See also SI Discussion S1, S2, S3, and Figure S2.

### Data Processing and Statistical Analyses

All data processing was conducted using R v 4.2.1 and RStudio v 2023.06.1 Build 438.^41^ The effect of temperature on V-Nase contribution to sample N fixation was tested using a generalized linear model (*glm*, with normal link function) with N-fixation activity corrected for V-Nase contribution (log_10_ BNF_N2_) and cryptogam type (moss or lichen) as covariables (Figure 2C–D, SI Tables S5–S6, Discussion S2, Figure S2). The effects of BNF and Mo were generalized using linear models, and variable selection was performed using Bayesian Information Criterion (BIC). Model formulations and output are detailed along the text and summarized in SI Tables S5–S8 (see also SI Discussion S2–S4).

### Pattern of Potential Enzyme Dominance in Individual Samples under Present and Past Deposition Regimes

Maps of potential V-Nase contribution to individual sample BNF were simulated using the relationship between measured %V-Nase, BNF rate, and Mo content in samples from this study, and the model Mo deposition data from Wong et al.^33^ and Wong et al.^42^ and model nitrogen deposition data from Tian et al.^43^ (see Relations 1 to 3 in Figure 3, SI Tables S7–S8, and Figures S3−S5).

**Figure 3:**
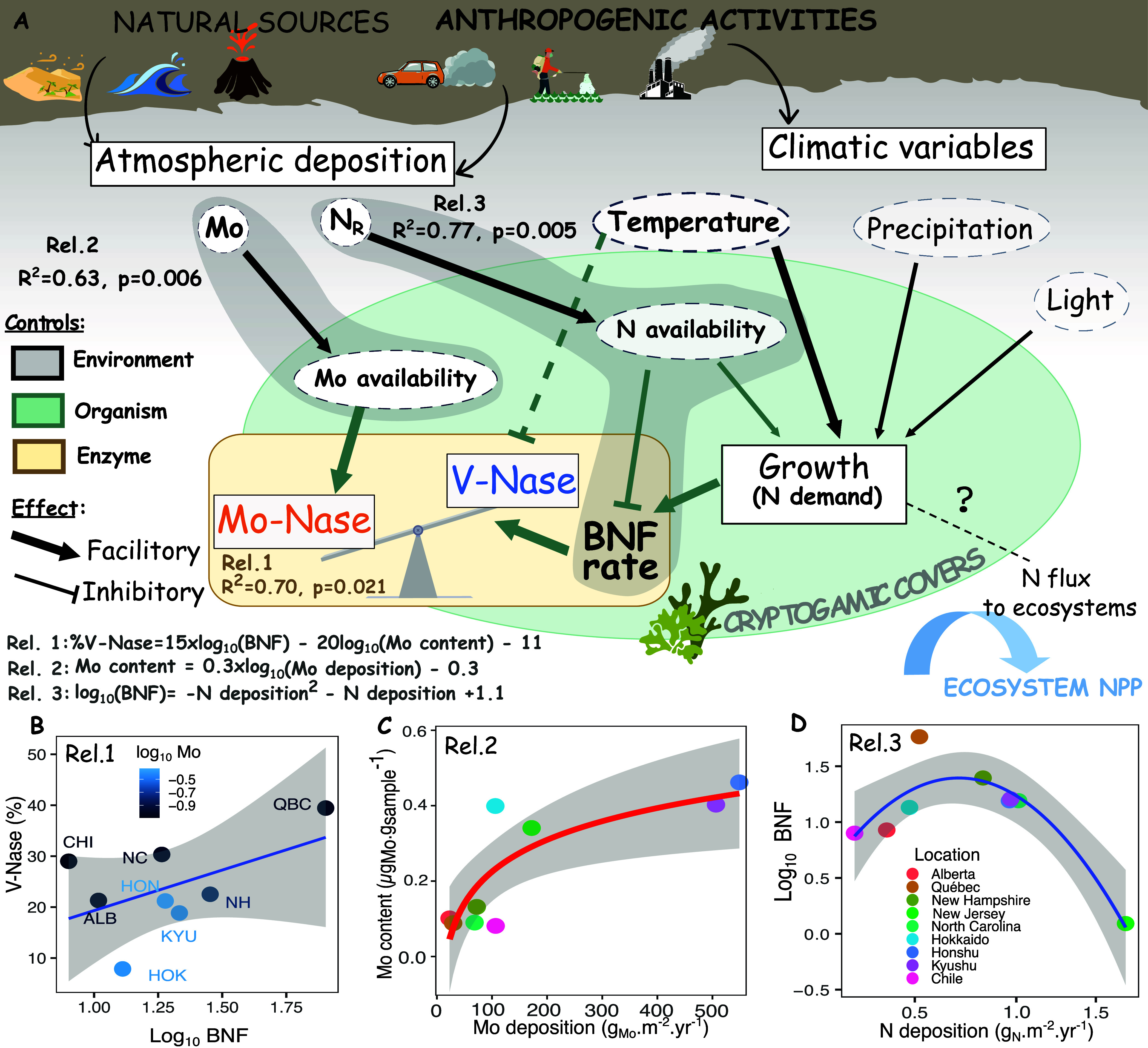
Conceptual schematic of environmental controls on V-Nase activity in cryptogamic covers (A) based on relationships with Mo and N (B–D). Panels B–D illustrate the relationships underlying Rel. 1 to 3, respectively. Relationship with only Mo deposition is presented in SI Table S8 (Rel.1.2). BNF rates are expressed in nmol_N2_ h^–1^ g_sample_^–1^.

Mo content and BNF rate for individual sample were estimated from model data using Relations 2 and 3, respectively, and then used to calculate V-Nase contributions in % total N_2_ reduction according to Relations 1.1 to 1.2 (Figure 3 and Table S8). Negative Mo predicted contents were floored to the minimal Mo content measured in this study (0.0075 ppm, ∼13% of values), and negative V-Nase contribution was floored to 0%, (∼4% of values), resulting in a trim of the extremum values (∼17% of all values).

Potential modern V-Nase contribution to sample BNF (Figure 4A) was calculated using model deposition data for Mo (including the anthropogenic contribution)^33^ and for nitrogen (using average over the last 5 years of available data, 2010–2015). Preindustrial estimates of sample V-Nase contribution (Figure 4B) were calculated using the natural Mo deposition model^42^ and the model nitrogen deposition data averaged between 1860 and 1865. Forested areas were delimited using the TERRA MODIS monthly NDVI 720 × 360 (resolution 0.5° × 0.5°) data set for July 2023.^44^ A threshold of 0.5 NDVI was used for the purpose of this estimation, which allowed us to include both closed canopy forested areas and low tree density areas where mosses and lichens are also found (i.e., alpine and arctic). Areas between 23.5° N and 23.5°S (tropical areas) were excluded from the results.

**Figure 4:**
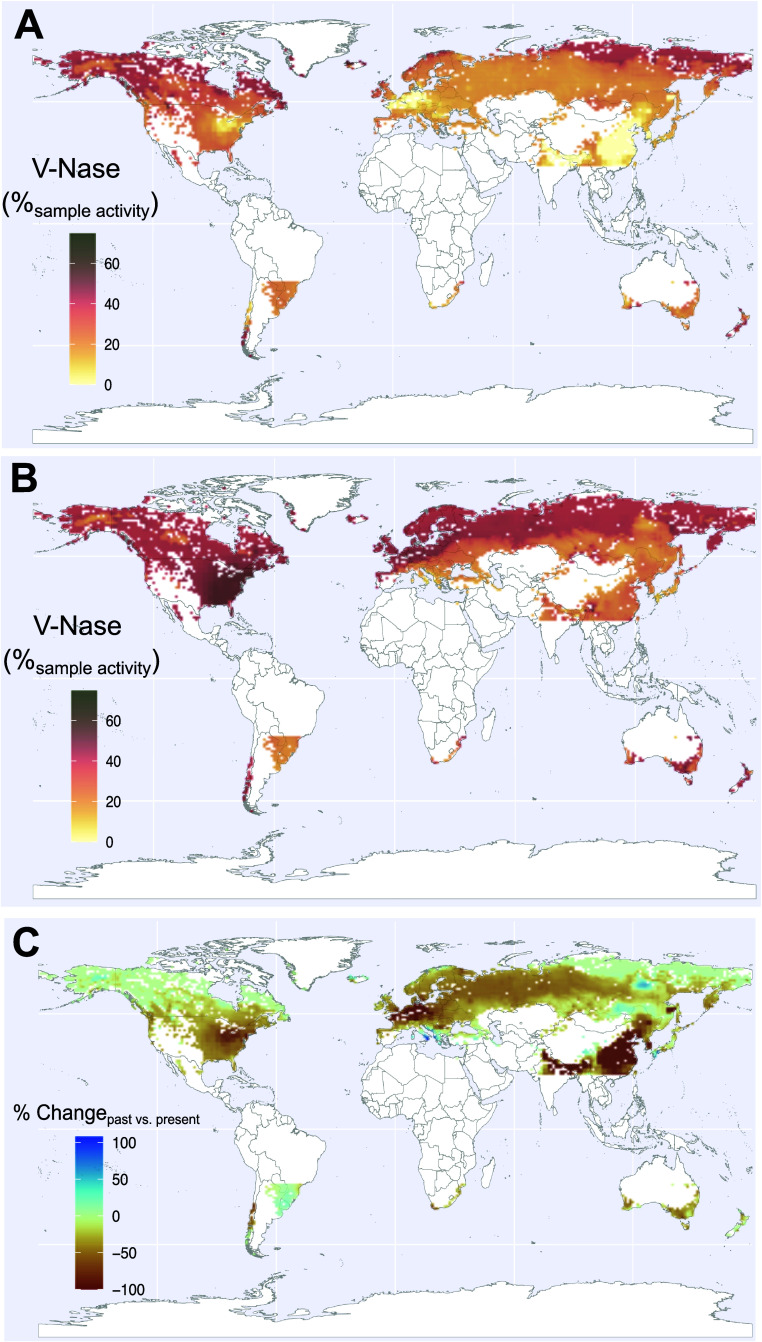
Regions of high potential V-Nase contribution to N fixation in individual samples of cryptogamic covers in extratropical forests. Current (A) and preindustrial (B) estimates are based on deposition of Mo^33,42^ and N deposition,^43^ and (C) shows the relative change (in % of change) between (A) and (B), with an average decrease in sample V-Nase contribution across extratropical forests of 30%. Maps A and B indicate the average proportion of V-Nase activity for BNF by an individual sample that can be expected for N-fixing samples collected at a chosen location.

Relative changes in V-Nase contribution to individual sample were calculated as the difference between present-day and past contribution normalized to past contribution for each grid cell (Figure 4C). Results excluding the V-Nase dependency on BNF are presented in the Supporting Information (SI Figure S5).

## Results and Discussion

### Widespread V-Nase Activity in Cryptogams from Global Extratropical Forests

Significant V-Nase activity (i.e., >CI_95%_ upper limit for the pure Mo-Nase isotopic signature) was detected in specimens from every collection location in South America, East Asia, and North America (Figure 1A). The occurrence of V-Nase activity in samples generating sufficient ethylene for LISARA (i.e., “valid”, >2 nmol_Ethylene_ g^–1^ h^–1^, 30 and 73% of tested samples for mosses and lichens, respectively) ranged from 28% (Hokkaido, Japan) to 94% (North Carolina). V-Nase activity was detected with an average 68 ± 20% positive sample across all locations and 79% across all valid samples (Figure 1A, Tables S1–S3). Fifteen out of the 28 genera tested demonstrated V-Nase activity in at least one sample, including some of the most abundant genera found in extratropical forests (*Pleurozium* sp., *Hypnum* sp., *Peltigera* sp., *Lobaria* sp.) and open areas (*Sphagnum* sp., *Stereocaulon* sp., and *Polytrichum* sp.) (Figure 1B).

Given the importance of N input by moss-associated cyanobacteria in high-latitude forests^8,9^ and previous reports of substantial V-Nase activity in boreal forest cyanolichens,^22^ we surveyed the cryptogams of Northeastern boreal forests more extensively than lower latitude regions (Figure 1C–D). Significant V-Nase activity (i.e., over 20% and up to 80% of total sample N_2_ fixation) was present in the four most abundant moss species within Eastern boreal forests, most notably in *Pleurozium schreberi* and *Hylocomium splendens*, which contribute as much as 50% of total N input across the circumboreal belt.^8,28,45^

V-Nase contributions in mosses are greater than or equal to activities measured for cyanolichens (Figure 1C–D). Notably, the distributions of V-Nase contributions to specimen BNF in each major cryptogam group in boreal forests and temperate areas are similar, with mosses and lichens averaging, respectively, ∼40% and ∼25% (Figure 1E). Additionally, two of the most ubiquitous genera in temperate forests, *Thuidium* sp. and *Hypnum* sp., show similar magnitudes of BNF rate to the species commonly found in boreal forests, such as*Pleurozium schreberi*,*Ptilium crista-castrensis*, or *Hylocomium splendens*­(see Figure 1B). Collectively, these results indicate that V-Nase is broadly used in lichens and bryophytes across extratropical forest ecosystems.

### A Global Mo Threshold in Forest Cryptogams

We examined how the contribution of V-Nase to specimen N fixation varies with Mo exposure (as estimated by sample Mo content) in the 12 most commonly surveyed species (i.e., with >10 samples measured) (Figure 2A). Most V-Nase activity was limited to samples with low Mo content (<0.5 μg_Mo_ g_sample_^–1^). Samples with V-Nase activity >40% of N_2_ fixation exhibited Mo content <0.2 μg_Mo_ g_sample_^–1^. This latter value is remarkably similar to the Mo threshold previously reported for boreal cyanolichens (<0.25 μg_Mo_ g_sample_^–1^),^22^ strongly indicating the existence of a general control of V-Nase by Mo availability in cryptogams.

We also measured the expression of V-Nase genes (*vnf*DG) on a subset of moss samples from two species collected at two boreal sites. We found variable levels of V-Nase gene expression, with particularly high expression in *Pleurozium schreberi*, an ubiquitous moss of the circumboreal belt (Figure 2B). Importantly, the few samples with no transcription activity had the highest Mo content (>0.1 μg_Mo_ g_sample_^–1^), consistent with the role of Mo as a control over V-Nase activity.

### Temperature Indirectly Modulates V-Nase Usage through N Demand

To investigate the effect of temperature on V-Nase activity, we conducted laboratory incubation experiments at four different temperatures (10, 15, 20, and 30 °C) using mosses and lichens collected from three areas of northeastern Canada. Incubation temperatures only influenced V-Nase activity at the lowest temperature (10 °C), which had the lowest contribution of V-Nase to N_2_ fixation (<20%, i.e., not significantly different from the pure Mo-Nase isotopic signal) (Figure 2C). This result was unexpected in view of prior *in vivo* and *in vitro* studies, which showed that V-Nase specific activity becomes progressively more competitive with that of the Mo-Nase when temperatures drop below 20 °C.^25,26^ However, we found a robust relationship (*p* < 0.001, *R* ^2^ = 0.33, *n* = 119, Figure 2D) between sample V-Nase contribution and the logarithm of BNF rate with an increase of roughly 10% in V-Nase contribution for each increase of one log_10_ unit in sample N_2_ reduction rate (see also SI Discussion S2 and Figure S2 for discussion on confounding effects and other methodological artifacts). The higher V-Nase contribution to individual samples when incubation temperatures are warmer than 10 °C could thus be the result of an indirect effect of warmer temperatures on growth, which would increase N demand and reliance on the V-Nase under limited Mo availability. For the relatively short duration of these experiments (24–48 h), if the amount of available Mo were to constrain the Mo-Nase pool, the organism would have to rely more on V-Nase (either newly synthesized or with a higher recruitment of existing enzymes) to fill its higher N requirement at warmer temperatures.

Interestingly, once the BNF rate is added into the statistical model for our data set, the cryptogamic group variable (i.e., mosses and lichens) is no longer significant. This indicates that the difference in sample V-Nase contribution between boreal mosses and lichens, as well as the spatial and temporal heterogeneity in V-Nase usage within each group, could be due to changes in both N demand and Mo availability (see SI Tables S5–S6 and Discussion S3). This new hypothesis is confirmed in all natural samples with available Mo content (*n* = 198) at the individual thalli level (*R* ^2^ = 0.19, *p* = 6.10–10, *n* = 193) and, to a lower extent, when aggregated at the site level (*R* ^2^ = 0.19, *p* = 0.036, *n* = 29), and at the large-scale location level (*R* ^2^ = 0.70, *p* = 0.021, *n* = 8) (see SI Table S7 and SI Figure S4A,B and SI Discussion S4). On these two last models, the *p*-value associated with log_10_BNF is over the traditional threshold (*p*-value ∼ 0.06). However, inclusion of log_10_BNF into the model results in significant model improvement (ΔAIC = −3, see SI Discussion S4).

### Mechanistic Controls on V-Nase Usage

At every scale investigated, the effect of increasing BNF rate on V-Nase contributions to sample BNF (+9–15%V-Nase log_10,BNF_^–1^, Table S7) is similar to the effect found in temperature manipulation experiments (+10%V-Nase log_10,BNF_^–1^, Figure 2D), which supports the findings that biologically fixed N demand could be a fundamental control on V-Nase activity. In all models, the coefficient associated with Mo is negative (i.e., inhibition of V-Nase contribution by Mo) and ranges from −12 to −21%V-Nase g_sample_^–1^ log_10_μg_Mo_^–1^ (Tables S7 and S8). Notably, there is no significant correlation between the V thallus content and V-Nase activity in our data (Figure S6). This is consistent with both (i) the absence of a direct regulatory pathway linking the V metal and V-Nase gene expression in N fixers, which contrasts with the well-characterized inhibition of V- and Fe-Nase gene expression by Mo metal,^46,47^ and (ii) with the higher abundance of V over Mo in a terrestrial environment,^29^ which likely prevents the occurrence of V limitation of BNF.

These results, together with the existing literature, allow us to propose a general framework for key environmental controls on V-Nase activity in cryptogamic covers (Figure 3).

V-Nase contribution to N fixation at the organism scale is influenced by both the BNF rate (i.e., the N demand unmet from other N sources) and the availability of Mo to the organism (i.e., representing the portion of fixed-N already supported by Mo-Nase)^22^ (see Figure 3A,B, eq 1). We generally expect Mo availability to the organism, and thus Mo-Nase synthesis and activity,^48^ to increase with higher Mo deposition flux. Similarly, increased N deposition fluxes reduce BNF rates by cryptogamic covers^49^ by supplying fixed-N to support growth demand. Regarding the effect of environmental parameters, higher temperatures, light exposure, and frequent precipitation can increase organism BNF rate^50^ due to their indirect effects on growth-driven N demand. Temperature also influences the relative efficiencies of the Mo- and V-Nases at the enzymatic level.^25,26^ As this effect was undetectable in our data, the effect of temperature on metabolic N demand may dominate the effect of temperature on isozyme specific activities. Organism N loss through gas formation (e.g., N_2_O) and the transfer of organic and inorganic N to the ecosystem^51^ are other pathways that can significantly increase N demand of the individual cryptogamic organism, favoring V-Nase usage by enhancing cellular Mo limitation. Finally, anthropogenic activities contribute additional, often codeposited Mo and N to existing natural sources.^33,42,52^ Anthropogenic activities may also increase the frequency and size of natural events (e.g., forest fires) through global changes in environmental change.

To validate our framework and mechanistically extrapolate our results beyond our survey, we investigated relationships between measured predictors for individual V-Nase contribution (sample Mo content and BNF rate) and available models of nutrient deposition (Mo, N). We found a significant relationship between average cryptogam Mo content at the location scale and the log of Mo deposition rate (*R* ^2^ = 0.68, *p* = 0.006, *n* = 9, Figure 3C and Rel. 2, Table S8), supporting the link between deposition and availability to the organism. We further identified a significant inverse quadratic relationship (*R* ^2^ = 0.77, *p* = 0.005, *n* = 8, Figure 3D and Rel. 3, Table S8) between log_10_BNF rate of specimens averaged at the location level and total N deposition as estimated from a nitrogen deposition model (2010–2015).^43^ This unimodal relationship suggests that low levels of N deposition could benefit N-fixers.^53^

### Spatial Estimates of V-Nase Contribution to Specimen BNF Identify High Potential V-Nase Usage

We use the proposed mechanistic framework (Figure 3A), regression relationships (Figure 3B–D, SI Table S8 Rel. 1–3), and Mo and N deposition spatial models^33,43^ to extrapolate potential V-Nase contribution to individual sample BNF activity across global extratropical forests under modern and preindustrial deposition conditions (Figure 4A–C). Maps of specimen-specific potential V-Nase usage that identify areas where *in situ* V-Nase activity is likely high (“hotspots”) can guide additional studies aimed at accurate quantifications of cryptogam BNF and its reliance on Nase heterogeneity for terrestrial N budgets. For this reason, we also include in our map areas of low vegetation cover (arctic and alpine steps) where mosses and lichens contribute to N cycling and where Mo and N depositions are likely to have a similar effect. Our dataset covers over 60% and 80% of the N and Mo deposition experienced by the selected areas, respectively, with a significant overlap between the two elements (SI Figure S3 and Table S9).

Under present-day Mo and N deposition conditions, potential V-Nase contribution to specimen-specific cryptogam N fixation in extratropical forests ranges from 0 to 50%. The map indicates that likely hotspots of V-Nase usage (>40%, red areas in Figure 4A,B) are mainly located in the Arctic area (northeastern and northwestern America and northeastern Asia).

Areas with likely no V-Nase activity (<15%, yellow areas in Figure 4A,B) are located in highly anthropized areas in upper western Europe, eastern USA, and East Asia. These areas are also where the influence of N deposition on V-Nase contribution is the most important in our model (SI Figure S5). More precise spatial estimates of V-Nase usage will also need to include the direct effect of other important variables for N demand and BNF rate beyond N deposition, such as temperature and humidity and the effect of increased CO_2_ concentration. These factors, which vary spatially and temporally, will likely modulate V-Nase contribution to BNF.

Our mechanistic approach enables evaluation of the potential extent to which anthropogenic activities have reduced the reliance of BNF on V since the onset of large-scale smelting industries (e.g., industrial revolution) by influencing elemental cycling and particularly by increasing Mo and N deposition in natural ecosystems. In the preindustrial period (< 1860), we estimate that V-Nase likely contributions to individual sample BNF were higher by 20% and 30% relative to current contribution in the boreal biome and global extratropical forests, respectively (Figure 4C). Decreases in V-Nase contributions in lower latitude ecosystems (by 50–100%) are consistent with the higher metal and nitrogen loads in temperate areas compared with boreal environments. Similar results were found with the Mo-only model (see SI Table S8, Rel. 1.1 and 1.2, Figures S4−S5).

### Environmental Implications

First, our data strongly suggest that new N inputs into ecosystems could be significantly underestimated given the global importance of cryptogamic species (including biocrust) to N fixation (13 to 49 Tg N per year),^6,7^ the widespread use of ARA for N flux quantifications, and the different calibration factors (*R* ratios) relating acetylene reduction to N_2_ fixation rate for isozymes.^18^ While large-scale V-Nase contribution remains to be demonstrated in other asymbiotic nitrogen-fixing substrates, the first estimates of alternative Nase activity in soil, litter, and deadwood from northeastern temperate forests show similar or higher V-Nase contribution to sample BNF than found here.^20^ This suggests that N fluxes from asymbiotic N fixers could also be underestimated.

Second, as BNF rate and Mo availability directly influence V-Nase usage in individual samples, which in turn influence their ethylene yield, our results call into question the reliability of using traditional ARA to quantify the effect of environmental variables on cryptogam BNF activity without systematically assessing samples under each experimental condition for the role of multiple Nase usage on BNF using ISARA. Our results also further support the idea that the sensitivity of BNF rate to Mo availability in nonsymbiotic N fixers is weaker than previously recognized, as V-Nase can act as a backup pathway for BNF. Similarly, isotope-constrained N cycle fluxes in areas with large populations of cryptogamic species should be reassessed as the isotopic composition of fixed N produced by the V-Nase is significantly lower than that from the Mo-Nase (by ∼5–6‰).^54^

Finally, the ubiquitous nature of V-Nase activity observed here suggests that V may play a more widespread role in nature than previously recognized.^55^ By highlighting new interactions of trace metal and nitrogen cycles that have important implications for terrestrial N budgets, our findings prompt updates to conceptual models and methods for N fixation to fully account for its enzymatic and metal heterogeneity and call for additional research on the roles of V in shaping terrestrial and marine biogeochemistry.

## Supplementary Material





## Data Availability

All data and R code used to produce the figures and tables are available at the following DOI address (10.6084/m9.figshare.25066190).

## Notes

Additional experimental, methodological, and statistical details, including summary tables of the sample (PDF)
All details about individual samples, including previously published data (XLSX)

## #

Centre de Recherche sur la Biodiversité et l’Environnement (CRBE), Université de Toulouse -CNRS-IRD-INPT Toulouse 31000, France (R.D.)

## †

Science and Technology Branch, Environment and Climate Change Canada, Montreal, QC 2E7, Canada (M.R.)
