# Supplementary material for: Global Role of Vanadium for Cryptogamic Nitrogen Fixation in Extratropical Forests
Source: Environ Sci Technol. 2026 Mar 10;60(12):9213–24. doi: 10.1021/acs.est.5c12982 (PMC13045013; doi:10.1021/acs.est.5c12982)
Supplement: Supplementary file 1 [file es5c12982_si_001.pdf]

Supplementary Information to: Global role of vanadium for cryptogamic nitrogen fixation in extratropical forests

Romain Darnajoux [1,2#*], Shannon J. Haynes[1], Marie Renaudin[3,4†], Nicolas Magain[5], Sessina Dani[2], Spencer Koonin[2], Jolanta Miadlikowska[6], Yoshitaka Uchida[7], Takamitsu Ohigashi[7], Diane Haughland[8,9], François Lutzoni[6], Jean-Philippe Bellenger[3,4], Xinning Zhang[1,2*]

[1]Department of Geosciences, Princeton University, Princeton, 08544 NJ USA

[2]High Meadows Environmental Institute, Princeton University, 08544 NJ USA

[3]Centre Sève, Université de Sherbrooke, Sherbrooke, J1K 2R1 QC Canada

[4]Département de chimie, Université de Sherbrooke, Sherbrooke, J1K 2R1 QC Canada

[5]InBioS Research Center, Université de Liège, 4000 Liège Belgium

[6]Department of Biology, Duke University, Durham, 27708 NC,USA

[7]Research Faculty School of Agriculture, Hokkaido University, Sapporo, 060-0808 Japan

[8]Alberta Biodiversity Monitoring Institute, University of Alberta, Edmonton, T6G 2E9 AB Canada.

[9]Department of Renewable Resources, University of Alberta, Edmonton, T6G 2H1 AB Canada.

Current addresses: # Centre de Recherche sur la Biodiversité et l'Environnement (CRBE), CNRS, Université de Toulouse, Toulouse, 31000, France.
† Science and Technology Branch, Environment and Climate Change Canada, Montreal, QC, H2Y 2E7, Canada.

*Corresponding authors
Email address: romain.darnajoux@utoulouse.fr, xinningz@princeton.edu

ORCID: 0000-0002-4996-0067 (R. Darnajoux) / 0000-0003-2763-1526 (X. Zhang)
0000-0002-5545-2130 (J. Miadlikowska) / 0000-0003-4849-7143 (F. Lutzoni)
0000-0002-0777-3693 (S. J. Haynes) / 0000-0002-6364-1740 (M. Renaudin)
0000-0003-1402-5041 (D. Haughland) / 0000-0001-6842-4561 (J.P. Bellenger)
0000-0003-4354-7842 (Y. Uchida) / 0000-0001-5409-9518 (N. Magain)

Keywords: Molybdenum, alternative nitrogenase, trace metal, temperature, cryptogamic covers, non-vascular phototrophs.

This document contains 32 pages numbered from S1 to S32, 6 Supplementary Figures numbered S1 to S6, and 9 Supplementary Tables numbered S1 to S9.

SUPPLEMENTARY METHODS

S1) Site descriptions

Eastern Canada*:* Six sites (S1-S6) were visited across a 600 km gradient in Québec (Canada) (Figure S1). The five northernmost sites are dominated by black spruce (*Picea marinana*), Jack pines (*Pinus banksiana*), most of which are covered with epiphytic lichens (*Evernia prunastris*, *Hypogenmia physodes*, *Usnea* sp., *Alectoria* sp. *Bryoria* sp.). The understory is composed of mosses (e.g., *Pleurozium schreberi* (Brid.) Mitt., *Ptilium crista-castrensis* (Hedw.) De Not., *Hylocomium splendens* (Hedw.) Schimp., *Dicranum polysetum* Swartz.), sporadic colonies of *Peltigera* (e.g., *P. aphthosa* s.l., *P.scabrosa*, *P. neopolydactylon* s.l.) and ericaceous species (*Rhododendron groenlandicum*, *Kalmia angustifolia*, *Gaultheria hispidula*) and ferns. These five northern sites fall along a strong gradient of metal deposition[1] and nitrogen deposition.[2] The southernmost site (S1) in Figure S1 and Figure 1C regroups samples collected at four sites south of Saint-Lawrence River (Mount Pinacle, Mount Royal in Montreal, Mont Orford, and a private land in the town of Sainte-Aurelie), located in the cold temperate maple forests, with occasional black spruce-balsam fir wood. Similarly, Site S3 regroups two black spruce-balsam fir wood sites (Laurentides roadside and Lac Bouchette) for simplicity.

All sites were sampled in June 2017. Site S4 was also visited in September 2015, in 2016 across the growing season (from end of May to end of September, 5 collection times), in June 2020, and in June 2022. Site S3 was also visited in June 2020 and June 2022, and Site 1 in June 2020. In June 2020, only lichens were collected in Site S1 and S3. In total for the six sites and across seven years, 228 samples were collected over 26 sub-sites, with 170 bryophyte samples and 58 lichen samples. Samples previously published in Darnajoux et al., 2019[3] were included in the SI Dataset S1 but excluded from Figure 1.

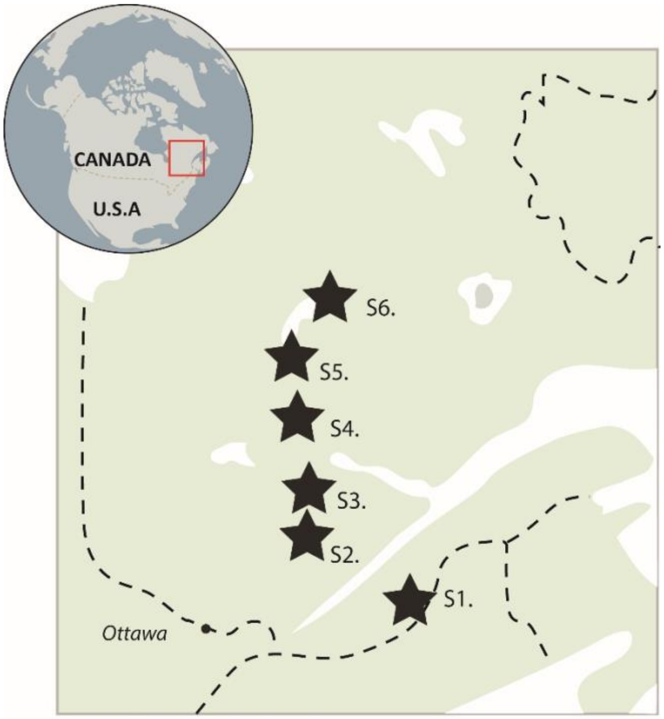

Figure S1: Location of sampling sites in Eastern Canada as presented in Main text Figure 1C and D.

Western Canada:

Alberta: Sampling sites are located in regions previously surveyed by the Alberta Biodiversity Monitoring Institute (www.abmi.ca) systematic monitoring program. The sites span an approximately 550 km latitudinal and 350 km longitudinal gradient, including montane, foothills, deciduous parkland and boreal forest ecozones. The sites included coniferous and mixed forests including Black spruce forests, Jack pine forests, Aspen-White spruce forests, and Pine-Poplar-Spruce forests, ranging in elevation from 635 to 1643 m. Sites varied from undisturbed to partially disturbed by resource extraction activities related to energy, forestry, and agriculture. A total of 9 sites were visited (one plot per site except at one site where two plots were sampled), with one site showing no significantly active samples, representing different canopy cover, moisture, and soil nutrient gradients for a total of 56 samples processed.

Eastern USA:

New Hampshire: The sole site visited in New Hampshire is located within the territory of Darthmouth college at Mount Moosilauke in the White Mountain. The site is a typical northern Appalachian Mountain Forest spanning elevation from 750 to 1460 meters, with ecozones covering mixed northern forest zone dominated by beech and maple, evergreen boreal zone dominated by red spruce and balsam fir, and Alpine tundra zone (open area above tree line with dominance of herbaceous vegetation). More details on the site can be found elsewhere.[4–6] Samples were collected at nine subsites along the South Peak loop for a total of 84 processed samples.

New Jersey: Three different forests were visited in New Jersey. The first one is the Institute of Advanced Studies Woods (Princeton, USA) located ~ 1 kilometer from the nearest large road and 2 kilometers from the closest city center. This site represents a secondary succession

chronosequence, originating from the rehabilitation of agricultural land that started as early as 1727 until 1940. The vegetation was dominated by maple, beech, or birch trees and the site was characterized by Horn 1975.[7] At this site in 2019, 32 samples were collected over 9 sub-sites, representing an example of temperate forest succession spanning ~80 to 300 years.

The second site is the Stony Ford Research Station, a research station maintained by the Ecology and Environmental Biology Department at Princeton University (https://eeb.princeton.edu/about-us/field-stations/stony-ford-research-station). This site is a typical broadleaves temperate forest established on a former farmland in 1967 and dominated by maples, oaks, and hickories.

The last site is the Watershed Institute Woods, a natural reserve and research station adjacent to several active and old farmlands (https://thewatershed.org/trails/). Samples were collected in deciduous and coniferous forests stands yielding 15 samples for processing.

No cyanolichens were found in any of the NJ forest sites.

North Carolina: Three sites were visited in the Blue Ridge Mountain region of western North Carolina. The first site is in the Highlands Biological Station, a research field station associated with the Western Carolina University in the city of Highlands (NC, USA) (https://highlandsbiological.org/). The site covers a diversity of forests, with an old-growth forest with hemlock and hardwood, hardwood forest, a bog garden, and a moss garden. The second site is located around the top of the Wayah bald (1615 m elevation) in the Nantahala National Forest (https://www.fs.usda.gov/recarea/nfsnc/recarea/?recid=48634). Samples were collected along different paths extending toward the Temperate broadleaf forests downhill to the bald. The final site is located at the Dry Falls waterfall, located 2 km away from the city center of Highlands in a

forested area. Samples at this site were collected on the waterfall cliff and on rocky outcrops along the path. A total of 106 samples were used in our experiment across the three sites.

Eastern Asia:

Hokkaido: Ten sites were visited in Hokkaido. Four sites were located in the far north, near the cities of Ottoineppu and Nakagawa, with two of these sites being the Nakagawa experimental forest and the Teshio experimental forest, two research stations maintained by Hokkaido University (https://www.hokudaiforest-e.jp/experimental-forests/). The two other sites were located near the Penkenai and Tenkita rivers. All the forest sites were mixed forest with Sasa bamboo-dominated understory. At the summits of two sites (Hakodake mountain, 1129 m, Penke mount, 709 m), mosses were found within a humid understory below short bushes within dry alpine vegetation (above tree line).

One site was located in the central part of the peninsula within the Taisetsuzan National Park. Samples were collected in the town of Sunkyo and along the trail from Sunkyo-Kurodake ropeway (670 m) to the mount Kurodake summit (1984 m). The vegetation extends from typically boreal mixed and spruce forest to alpine tundra above the tree line.

Two other sites were found in the extreme east of the Hokkaido peninsula, one site within the Shiretoko National Park and the other near Shibetsu, a forestry village.

The three final sites are located in the southern part of the peninsula, two in the remote Hidaka mountains (one near the Iwachishi river and another near Petegari mount) and one at the Hakodate observatory in the town of Hakodate.

Honshu: The two sites visited are located in the vicinity of mount Ontake, in the Nagano prefecture. At the first site, samples were collected along the trail from the Mitake Tourist center to the top of mount Ontake and extended from a mixed forest to an alpine tundra above the tree line. The second site was located along the Kiso Road from the Otaki Visitor Center to the Mitake Tourist Center, and was composed of temperate mixed forest.

Kyushu: One site comprising six sub-sites was sampled in the vicinity of Mount Raizan, along the trail from the Mizunashi entrance to the mount Ibara. The vegetation is a humid warm temperate deciduous forest with abundant mosses covering the floor, the vegetation, and outcrops.

<u>South America</u>:

Chile: Six sites in South Macroregion of Chile were sampled. They are located within the Neotropic temperate broadleaf and mixed forests biome (the Valdivian temperate forest ecoregion) extending from 130 m to 1500 m in altitude. Lichens were collected in Parque Nacional Puyehue (Region de los Lagos), Reserva Nacional Mocho-Choshuenco and Monumento Nacional Alerce Costero (Region de los Rios), and Parque Nacional Nahuelbuta and Reserva Nacional Malalcahuello (Region de la Araucaria). Lichen collections were obtained from the primary and secondary forests, e.g., Notofagus forest, mixed Valdivian forest, and Araucaria forest, and Andean grassland and scrub vegetations.

S2) Estimation of V-Nase contribution for individual samples

Within one month of sample collection, 0.08-0.3 gDW of dried sample were put in 40 mL borosilicate glass vial (VWR, USA). Samples were re-wetted to saturation using 2 to 5 mL of DI water and the excess water was carefully discarded after ~ 15 minutes. Samples were left to acclimate for ~ 10-12 hrs overnight at room temperature before performing the Acetylene Reduction Assay (ARA) incubation. The next morning, vials were closed with open-top caps fitted with a 24 mm PTFE/Silicon septum (Supelco, Sigma-Aldrich, St. Louise, USA) and ~ 10% v/v of the headspace was replaced by acetylene freshly prepared from the addition of DI water to calcium carbide (technical grade, < 10 mm, Sigma Aldrich, St. Louise, USA). Acetylene reduction assay incubation lasted for 24 h in laboratory condition with constant light. After these 24hr periods, headspace (15-18 mL) was removed from the vials and transferred first to a 12 mL brown glass serum vial (Wheaton, Fisher Scientific) fitted with a 20mm blue butyl septa (Bellco Glass) for ethylene $\delta^{13}C$ isotopic analyses, then to a 5mL Exetainer® tube for ethylene concentration quantification. On the day of the incubation, several aliquots of the source acetylene were saved in a 12 mL brown glass vial or as a 10% v/v dilution in a 27 mL serum bottle, both fitted with blue butyl septa. These reference samples were kept in the dark until further processing

The concentration of ethylene within ARA incubations of each sample was first determined using a GC-FID (GC-8A, Shimadzu, Japan) equipped with a Supelco® custom packed column (1/8" x 2.1, mm stainless steel column packed with 80/100 HayeSep N resin). Then, ethylene isotopic values of the samples, expressed as $\delta^{13}C_{ethylene,sample}$, were obtained using a home-made pre-concentration system connected to a commercial gas chromatograph with a combustion reactor and a Thermo Scientific™ DeltaV Isotope Ratio Mass Spectrometer (PreCon-GC-C-IRMS), as described in Haynes et al., 2022.[8]

Reference samples for the source acetylene in ARAs were first converted into ethylene using ARA incubations on a set of two double-deletion mutant bacteria (strains CA70.1 and CA11.70, later referred to as Mo-only and V-only mutant, respectively), conducted in triplicate and as described earlier.[8] The resulting ethylene $\delta^{13}C$ values are referred to as $\delta^{13}C_{ethylene,Mo}$ and $\delta^{13}C_{ethylene,V}$ for the Mo- and V-Nase mutant, respectively.

V-Nase contribution in samples from 2015-2017 were calculated according to Zhang et al., 2016[9] using direct measurement of the $\delta^{13}C_{acetylene}$ (see Eq. S1.).

$$\%VNase_{AR} = \frac{({}^{13}\varepsilon_{AR,Mo} - {}^{13}\varepsilon_{AR,sample})}{({}^{13}\varepsilon_{AR,Mo} - {}^{13}\varepsilon_{AR,V})} \qquad \text{Eq. S1}$$

With ${}^{13}\varepsilon_{AR,i}$ the difference between the isotopic signal of ethylene produced during ARA ($\delta^{13}C_{ethylene,i}$) and the isotopic signal of the source acetylene ($\delta^{13}C_{acetylene}$) for the metal i.

V-Nase contributions to acetylene reduction in samples from 2019 to 2020 were calculated using one anchor point (usually $\delta^{13}C_{ethylene,Mo}$), and the average scaling factor values found across three different pairs of bacterial mutant strains (*Azotobacter vinelandi*, *Rhodopseudomonas palustris*, and *Anabaena variabilis,* $\Delta^{13}\varepsilon_{Mo-V} = {}^{13}\varepsilon_{Mo} - {}^{13}\varepsilon_{V} = 6.0$ ‰ , SD =0.6 ‰ , n=8, details in Haynes et al 2022) according to Eq. S2.

$$\%VNase_{AR} = \frac{(\delta^{13}C_{ethylene,Mo} - \delta^{13}C_{ethylene,sample})}{(\Delta^{13}\varepsilon_{Mo-V})} \qquad \text{Eq. S2}$$

Finally, V-Nase contribution to acetylene reduction in samples from 2021 and 2022 were directly determined using the mutant reference values $\delta^{13}C_{ethylene,Mo}$ and $\delta^{13}C_{ethylene,V}$ according to Eq. S3.

$$\%VNase_{AR} = \frac{(\delta^{13}C_{ethylene,Mo} - \delta^{13}C_{ethylene,sample})}{(\delta^{13}C_{ethylene,Mo} - \delta^{13}C_{ethylene,V})} \qquad \text{Eq. S3}$$

All contribution of V-Nase toward acetylene reduction were then converted in contribution of V-Nase toward BNF using the formula from Zhang et al. 2016 (Eq. S4).

$$\%VNase_{BNF} = \frac{\left(\frac{\%VNase_{AR}}{R_V}\right)}{\left(\frac{\%VNase_{AR}}{R_V} + \frac{(1-\%VNase_{AR})}{R_{Mo}}\right)} \qquad \text{Eq. S4}$$

With $R_{Mo} = 4$ and $R_V = 2$, being the calibration factor between acetylene and dinitrogen reduction for Mo- and V-Nase, respectively.[10]

S3) RT-qPCR conditions

Samples were ground with a mortar and pestle and total RNA was extracted using the NucleoSpin RNA Plant and Fungi kit (Macherey-Nagel). Residual DNA was digested with the rDNase Set (Macherey-Nagel). Extracted RNA was converted into cDNA with the LunaScript SuperMix kit (New England BioLabs) and the cDNA product was subsequently cleaned with the QIAquick PCR Purification kit (Qiagen). Then, the *vnf*DG region and cyanobacterial 16S rRNA gene segments (used as the housekeeping gene) were amplified by qPCR with the primer pairs VN 60F/VN 807R[11] and CYA359F/CYA781Ra [12] respectively. The qPCR was carried out in triplicates and each 20 µl qPCR reaction included 10 µl of PerfeCta SYBR Green FastMix (Quantabio), 2 µM of forward and reverse primers, and 2 µl of cDNA template. Negative and positive (DNA isolated from *Azotobacter vinelandii* OP and *Nostoc* 232) controls were used for each qPCR run. The two target regions were amplified in a CFX Connect Real-Time PCR System (Bio-Rad) with the following procedure: initial denaturation at 95°C for 3 min, followed by 38 cycles at 95°C for 45s, and 56°C for *vnf*DG/60°C for 16S rRNA for 1 min. The Ct was calculated by the device's software by following the evolution of fluorescence during time and, for each sample, the *vnf*DG fold change was manually calculated with the $2^{-\Delta\Delta Ct}$ method (see Schmittgen & Livak, 2008). Samples displaying negative ΔCt (i.e., difference between *vnf*DG Ct and 16S rRNA Ct) were considered as not containing the *vnf* gene and discarded from further analysis.

Table S1: Summary of sample locations

Location	Country	Sampling year(s)	Number of sites	Number of sub-sites	Latitude		Longitude		Altitude	
					**min**	**max**	**min**	**max**	**min**	**max**
Alberta	Canada	2022	9	10	50.5472	55.54186	-117.369	-114.037	635	1651
Québec	Canada	2015-2016-2017-2020-2022	9	38	45.02387	51.1339	-73.969	-70.3685	350	767
New Hampshire	USA	2021	1	9	43.99916	44.01948	-71.8387	-71.8201	850	1412
New Jersey	USA	2019-2022	3	7	40.32631	40.35522	-74.783	-74.667	62	64
North Carolina	USA	2022	3	13	35.05327	35.1892	-83.5889	-83.1845	1026	1450
Hokkaido	Japan	2019	10	21	41.75708	44.85972	140.6985	145.1873	50	1737
Honshu	Japan	2019	2	4	35.82608	35.87604	137.4995	137.5524	1042	2111
Kyushu	Japan	2019	1	7	33.48125	33.48299	130.2609	130.2638	570	670
Central Chile	Chile	2017	6	10	-40.7847	-37.8195	-73.4565	-71.5429	130	1500
**TOTAL**			**44**	**109**						

Table S2: Summary of sample functional groups for each geographic location. Moss and lichen samples are further classified into functional types (acrocarpeous vs. pleurocarpeous, bimembered, i.e., only fungi and cyanobacteria, vs. trimembered, i.e., fungi, green algae, and cyanobacteria) or evolutionary group (*Sphagnum* sp., Liverwort).

**Geography**	**Bryophytes**	*Acrocarps*	*Liverwort*	*Pleurocarps*	*Sphagnum*	**Lichen**	*Bimembered*	*Trimembered*	**Total**
Alberta	28	0	0	28	0	28	15	13	56
Québec	170	5	2	156	7	122	78	44	292
New Hampshire	78	29	3	42	4	6	5	1	84
New Jersey	55	40	0	15	0	0	0	0	55
North Carolina	82	11	13	53	5	24	18	6	106
Hokkaido	21	1	2	18	0	32	18	14	53
Honshu	18	4	0	13	1	20	11	9	38
Kyushu	17	0	1	16	0	5	5	0	22
Central Chile	0	0	0	0	0	40	16	24	40
**TOTAL**	**469**	**90**	**21**	**341**	**17**	**277**	**166**	**111**	**746**

Table S3: Summary of LISARA incubations of bryophyte samples. N-fixing samples showed significant ethylene production (>2 ppmv above background). LISARA samples are producing >10 ppmv ethylene (> 2 $nmol_{Ethylene}.g^{-1}.hr^{-1}$). Positive samples are those with > 15% V-Nase contribution to N_2 fixation.

**Geography**	**Dominant biome**	**Sample tested**	**N-fixing samples** *number (%[a])*	**LISARA samples** *number (%[a])*	**Methods**	**Positive** *number (%[b])*	**Negative** *number (%[b])*
Alberta	Boreal	28	19 (68)	10	Eq. S2-S3	4 (40)	6 (60)
Quebec	Boreal	170	125 (74)	84	Eq. S1-S2-S3	74 (88)	10 (12)
New Hampshire	Temperate	78	12 (15)	3	Eq. S3	3 (100)	0 (0)
New Jersey	Temperate	55	38 (69)	11	Eq. S2-3	10 (91)	1 (9)
North Carolina	Temperate	82	47 (57)	24	Eq. S3	23 (96)	1 (4)
Hokkaido	Boreal	21	9 (43)	3	Eq. S2	3 (100)	0 (0)
Honshu	Temperate	18	10 (55)	3	Eq. S2	3 (100)	0 (0)
Kyushu	Temperate	17	4 (24)	2	Eq. S2	1 (50)	1 (50)
Chile	Temperate	0	-	-	-	-	-
Sub-total	Boreal	219	153 (70)	97 (44)		81 (84)	16 (16)
	Temperate	250	111 (44)	43 (17)		40 (93)	3 (7)
**TOTAL**		**469**	**264 (56)**	**140 (30)**		**121 (86)**	**19 (14)**

[a]Percentage of N-fixing and LISARA samples relative to total tested samples. [b]Percentage of positive and negative samples relative to valid LISARA samples.

Table S4: Summary of LISARA incubations of cyanolichen samples. N-fixing samples are samples showing significant ethylene production (>2 ppmv above background), LISARA samples are producing >10 ppmv ethylene (> 2 $nmol_{Ethylene}.g^{-1}.hr^{-1}$). Positive samples are those showing > 15% V-Nase contribution to N_2 fixation.

Geography	Dominant biome	Sample tested	N-fixing samples *number (%[a])*	LISARA samples *number (%[a])*	Methods	Positive *number (%[b])*	Negative *number (%[b])*
Alberta	Boreal	28	28 (100)	26 (93)	Eq. S3	14 (54)	12 (46)
Quebec	Boreal	122	122 (100)	110 (90)	Eq. S1-S2-S3	93 (85)	17 (15)
New Hampshire	Temperate	6	6 (100)	6 (100)	Eq. S3	5 (83)	1 (17)
New Jersey	Temperate	0	-	-	-	-	-
North Carolina	Temperate	24	24 (100)	23 (96)	Eq. S3	21 (91)	2 (9)
Hokkaido	Boreal	32	23 (72)	15 (47)	Eq. S2	2 (13)	13 (87)
Honshu	Temperate	20	18 (90)	11 (55)	Eq. S2	8 (40)	3 (60)
Kyushu	Temperate	5	4 (80)	3 (60)	Eq. S2	1 (33)	2 (67)
Chile	Temperate	40	20 (50)	9 (23)	Eq. S1	5 (56)	4 (44)
Sub-total	Boreal	182	147 (81)	151 (83)		109 (72)	42 (28)
	Temperate	95	72 (76)	52 (55)		40 (77)	12 (23)
**TOTAL**		**277**	**245 (88)**	**203 (73)**		**149(73)**	**54 (27)**

[a]Percentage of N-fixing and LISARA samples relative to total tested samples. [b]Percentage of positive and negative samples relative to valid LISARA samples.

SUPPORTING DISCUSSION

S1) Metal analyses quality control and sample validation.

Peach leaves (Certified Reference Material) results show high reproducibility for Mo (RSD < 10%) with a systematic underestimation of metallic element (Mo, V, Fe, Al) of approximately 40%. This result is consistent with the use of HNO_3 only in our digestion process, while the NIST procedure for Mo includes HNO_3 digestion together with HF and $HClO_3$, which will solubilize Si-bound and other recalcitrant minerals. Hence, we interpret Mo data as representing the more labile fraction of Mo. As reproducibility was good ($RSD_{interrun}$ < 10%), samples remain comparable.

All but one procedural blank was lower than instrumental detection limits (0.017 ppb), with low RSD (0.003 ppb). One out of the nine blanks showed clear sign of metal contaminations (Mo, V, W, Fe, no Al). Using the presence of W (found at a higher concentration than Mo in the contaminated blank) as a marker for contamination, we estimate that < 10% of the samples (~ 20 out of 250 analyzed) could have been subject to some level of contamination, with > 60% of them found in a single geographical location (northeastern Canada). Similarly, results from the 32 microwave-processed samples were batch-corrected for contamination (based on blank value) and the accuracy of the correction was verified using the Peach leave standard with satisfactory post-correction accuracy (116%). To reduce the overall impact of these contaminations in our statistical analyses, we used the median and median absolute deviation as robust estimators of centrality and spread. We conclude that this contamination, while possibly impacting the average Mo value of some sites, did not impact the detection of a Mo threshold and our interpretation of the data.

S2) Influence of spurious correlation between V-Nase contribution and BNF rate.

Because N-fixation activity corrected for V-Nase contribution ($BNF=BNF_{N2}$, Main text Eq. 5) was calculated using V-Nase contribution data, it is possible that part of the explained variance in the model results from sheer spurious correlation.[14] To evaluate this scenario, we used a bootstrap approach (n=1000) on data simulated using the means and averages from the $\log_{10}$ of BNF_{N2} (mean=1.5, sd=1) and V-Nase contribution (mean=15%, sd=10%) and assuming a normal distribution. The results only slightly deviated from the null hypothesis (H0: no spurious correlation effect), with the average slope slightly different from zero (mean= 0.5%, sd=0.05%, $p= 1.10^{-16}$, n=1000, See Fig. S3), and 6% of the number of events showing significant slope at 95% confidence level (instead of 5% in the case of the null hypothesis). Finally, 99% of all simulated slopes values were below 4.5% (compared to the slope of 11 %V-Nase per log unit from our data). These results allow to ruled out major contribution of spurious correlation.

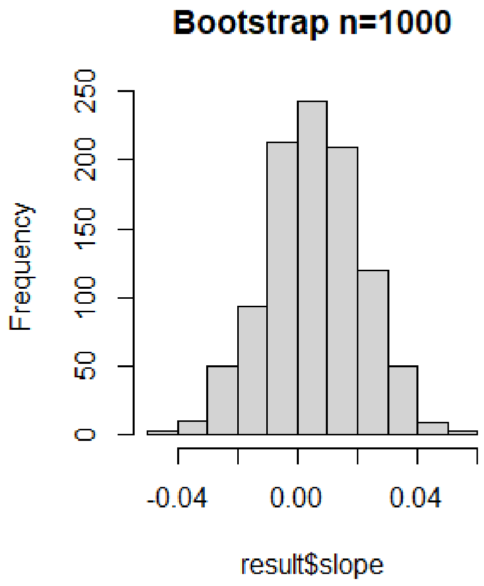

Figure S2: Statistical distribution of the slope between V-Nase contribution and the corrected BNF activity ($\log_{10}$ BNF_{N2} calculated according to Eq. S5) from a bootstrap simulation (n=1000) using simulated normal data to evaluate the magnitude of the spurious regression between BNF correction accounting for V-Nase contribution.

S3) Statistical assessment of interrelated effects of temperature, cryptogam group, and BNF activity variables on V-Nase contribution in temperature manipulation experiments

N-fixation activity generally increases with temperature and is higher in cyanolichens than in mosses (Table S5). Hence, there is a strong confounding effect (inter-correlation) present in the dataset that can influence the output of the statistical models. To constrain the influence of each variable, we use a backward – forward variable selection approach, evaluating the significance of individual variables within the general model using the *anova* function on general linear model outputs (*glm*) (Table S6). When a variable was found to not significantly improve the model (based on a Chi-test on deviance), it was excluded. We retained the most parsimonious model (i.e., the one with the least number of variables and highest deviance) using the minimum Schwartz' Bayesian Criterion (BIC) value approach, which accounts for the number of degrees of freedom of the model and the sample size. Our results show that the measured difference in BNF rate between cryptogamic groups (lichen vs. bryophytes) can fully account for the observed differences in V-Nase contribution between the same groups (ΔBIC = 4.5, p =0.965, Table S6), hence removing the significant effect of such grouping on V-Nase contribution. Similarly, the inclusion of Temperature in addition to BNF slightly improve the overall model (p =0.023), but the effect is mostly due to the additional degree of freedom used (ΔBIC = 4, Table S6), so we did not retain it in the final model formulation.

S4) Variables selection of predictive model for the identification of V-Nase activity hotspot in cryptogamic cover.

During the variable selection for our model, Relation 1 (see Table S7 and S8) shows global significance ($p = 0.02$) yet, one of the variables ($\log_{10}$ BNF) was not found to be significant ($p=0.06$). A Bayesian approach relying on the Bayesian Information Criterion (BIC), a highly parsimonious criterion for variable selection, indicates that including $\log_{10}$ BNF provide a significant reduction in the deviance of the model, with a ΔBIC of -3 (see Table S8). This contradiction of traditional inference (p-value based) and Bayesian inference (BIC-based) for model variable selection has been discussed elsewhere[15] where the authors advice to investigate effect size of such *confusing variable* and use other argument to conclude. In our specific case, the closeness of p-value to the traditional threshold (0.06 vs 0.05), the significant improvement in the model deviance (ΔBIC = -3, Table S8) and adjusted-R^2 (0.7 vs. 0.47, Table S8) upon adding the variable, the global significance of the model ($p=0.02$), and the similar effect size of $\log_{10}$ BNF in the model (15 ± 6%.$\log_{10}$ BNF^{-1}), the dedicated experiment (10 ± 6 %.$\log_{10}$ BNF^{-1}, Main text Figure 2D), and across the highly significant model that use the whole dataset (9 ± 2 %.$\log_{10}$BNF^{-1}, $p = 6.10^{-10}$, Table S7) strengthen our case to retain $\log_{10}$ BNF for our spatial estimate of V-Nase activity. We nevertheless provide a Mo-only model for comparison in SI (Fig. S4), which support all the conclusion made in the Main text discussions.

Table S5: Result of the linear model linking BNF rate (BNF, log$_{10}$ transformed BNF$_{N2}$ in nmol$_{N2}$.hr^{-1}.g$_{sample}$$^{-1}$**) to temperature (TEMP, categorical) and cryptogamic groups (CRYPTO, categorical, i.e., lichens or moss) (n=91).**

Model : BNF~TEMP+CRYPTO

**Variables[1]**	**Estimates**	**Std. Error**	**t-value**	**Pr(>|t|)**	**Significance**
Intercept (as TEMP_10 / MOSS)	-0.79	0.10	-7.65	2.39.10-11	***
TEMP_15	0.48	0.15	3.12	0.00248	**
TEMP_20	0.42	0.12	3.44	0.000915	***
TEMP_30	0.54	0.12	4.51	2.03.10^{-05}	***
LICHEN	1.47	0.09	15.56	<2.00.10^{-16}	***

[1]No significant interaction was identified between cryptogamic group and temperature.

Table S6: Model selection[a] for the effect of temperature (TEMP, categorical), cryptogamic group (CRYPTO[b], categorical), and nitrogenase activity (BNF[c], continuous, $\log_{10}$ $nmol_{N2}.hr^{-1}.g_{sample}^{-1}$) on V-Nase contribution (LISARA, % N_2 fixation) for the temperature manipulation experiment (Figure 2C and D).

Sequential model comparison	Variable tested	DF (value and Δ)	Deviance (value and Δ)	BIC (value and Δ)	p value (>chi)	Interpretation
Backward model[a]						
LISARA ~ 1		90	10882			
LISARA~BNF+TEMP+CRYPTO		85	6676.3	680.7		
LISARA~BNF+TEMP		86	6770.4	677.5		
	**CRYPTO**	-1	-94.1	3.2	0.274	**Non significant**
LISARA~BNF		89	7519.3	673.5		
	**TEMP**	-3	-748.9	-4	**0.023**	**Significant**
LISARA~TEMP		87	8961.82	693.5		
	**BNF**	-1	-2190.8	16	**$1.3.10^{-7}$**	**Highly significant**
Forward model						
LISARA~BNF		89	7519.3	673.5		
LISARA~BNF+CRYPTO		88	7519.1	678.0		
	**CRYPTO**	-1	-0.2	4.5	0.962	**Excluded**
Final model retained						
**LISARA~BNF**		152	10405	1101.0		**Selected**

[a]No interaction between variables were identified in the initial model. [b]Cryptogam group codes in Table S2, Data File S1. [c] BNF_{N2} of Main text Eq. 2.

TABLE S7: Linear model between V-Nase contribution (% to N_2 fixation), Mo content (µg Mo g_{ODW}^{-1}), and BNF rate[a] ($nmol_{N2}.hr^{-1}.g_{sample}^{-1}$) at various spatial levels of aggregation.

AGGREGATION LEVELS	MODELS	STATISTICS				
		**Adj-R^2**	**F-statistic**	**DF**	**p-value**	**Significance**
**Individual samples (all samples, n=193)**	*V-Nase ~ log_{10} BNF Rate + log_{10} Mo content*	0.19	23.71	2;194	**6.10^{-10}**	***
			Coefficient	*SE*	*p-value*	
		Intercept	2	4	0.61	
		log_{10} BNF Rate	9.1	1.7	**4.10^{-7}**	***
		log_{10} Mo content	-17	4	**$2.3.10^{-6}$**	***
		**Adj-R^2**	**F-statistic**	**DF**	**p-value**	**Significance**
**Site (n=29)** [b]	*V-Nase ~ log_{10} BNF Rate + log_{10} Mo content*	0.19	3.87	2;23	**0.036**	*
			Coefficient	*SE*	*p-value*	
		Intercept	0	7	0.87	n.s.
		log_{10} BNF Rate	11	5	0.055	.
		log_{10} Mo content	-12	5	0.055	.
		**Adj-R^2**	**F-statistic**	**DF**	**p-value**	**Significance**
**Location (n=8)** [b] **Rel. 1**	*V-Nase ~ log_{10} BNF Rate + log_{10} Mo content*	0.70	9.32	2;5	**0.021**	*
			Coefficient	*SE*	*p-value*	
		Intercept	-11	9	0.29	n.s.
		log_{10} BNF Rate	15	6	0.06	.
		log_{10} Mo content	-20	6	**0.018**	**

[a]BNF rate as BNF_{N2} of Main text Eq. 2. [b]Inclusion of log_{10} BNF Rate in the Site and Location model provide the lowest BIC and lowest residual deviance despite p value over the traditional 0.05 threshold (See Discussion S4).

Table S8: Linear relationships at location level used to estimate contributions likelihood of V-Nase in forested areas. V-Nase contribution as % N_2 reduction, Mo content in $\mu g_{Mo}.g_{sample}^{-1}$, BNF rate in $nmol_{N2}.hr^{-1}.g_{sample}^{-1}$)

MODELS	DESCRIPTION	STATISTICS						
		**Adj-R²**	**F-statistic**	**DF**	**p-value**	**Significance**	**Deviance**	**BIC**
Main text[a] Rel. 1	*V-Nase ~ log_{10}(BNF Rate) + log_{10}(Mo content)*	0.70	9.32	2;5	**0.021**	*	128.48	53.2
			Coefficient	*SE*	*p-value*			
		Intercept	-11	9	0.29	n.s.		
		log_{10}(BNF Rate)	15	6	0.06	.		
		log_{10}(Mo content)	-20	6	**0.018**	**		
		**Adj-R²**	**F-statistic**	**DF**	**p-value**	**Significance**	**Deviance**	**BIC**
SI Rel. 1.2	*V-Nase ~ log_{10}(Mo content)*	0.47	7.24	1;6	**0.036**	*	275.26	57.2
			Coefficient	SE	p-value			
		Intercept	8	6	0.26	.		
		log_{10}(Mo content)	-21	8	**0.036**	*		
		**Adj-R²**	**F-statistic**	**DF**	**p-value**	**Significance**		
Main text Rel. 2	*Mo content ~ log_{10}(Mo deposition)*	0.63	14.67	1;7	**0.006**	**		
			Coefficient	SE	p-value			
		Intercept	-0.34	0.15	0.06	.		
		log_{10}(Mo deposition)	0.28	0.07	**0.006**	**		
		**Adj-R²**	**F-statistic**	**DF**	**p-value**	**Significance**		
Main text Rel. 3	*log_{10}(BNF rate) ~ N deposition²+ N deposition*	0.77	14.69	2;6	**0.005**	**		
			Coefficient	SE	p-value			
		Intercept	1.1	0.07	**5.10^{-6}**	***		
		Ntot deposition	-1.3	0.6	**0.035**	*		
		Ntot deposition²	-1.0	0.2	**0.003**	**		

[a]Inclusion of log_{10} BNF Rate in the ISARA predictive model provide the lowest BIC and lowest residual deviance despite p-value over the traditional 0.05 threshold (See Discussion S4).

Table S9: Coverage of our sampling campaign with respect to global deposition model of Mo[16] and N[17] in extratropical forest ecosystems.

		This study range ($\mu g.m^{-2}.yr^{-1}$)	Model range ($\mu g.m^{-2}.yr^{-1}$)	% of ETF outside of range
Mo deposition rate	**Min**	15.8	1.34	17
	**Max**	548.8	2250	5
N deposition rate	**Min**	0.158	0.009	12
	**Max**	1.20	3.89	26
Overlap of Mo and N deposition rate	**Min**	-	-	27
	**Max**	-	-	13
	**Sum**	-	-	40

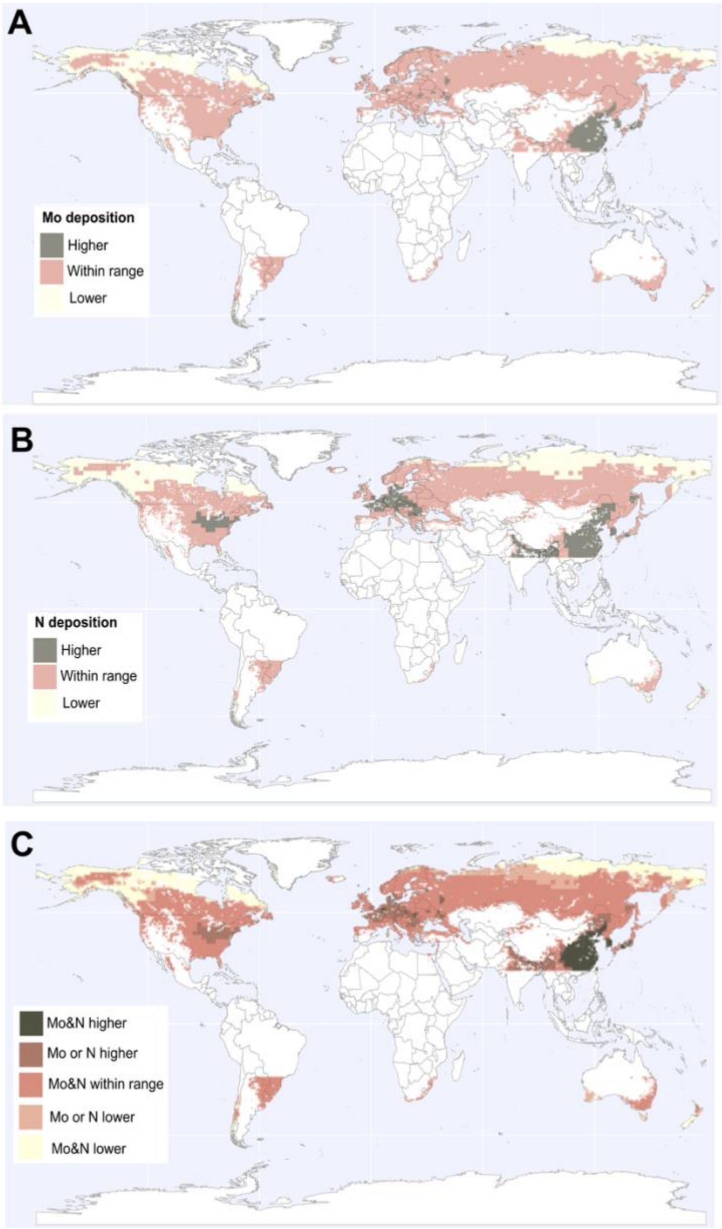

Figure S3: Relevance of our sampling coverage regarding atmospheric deposition of molybdenum (A) and nitrogen (B). Panel (C) overlaps both Mo and N deposition relevance, highlighting that most extratropical forest deposition rates are within our sampling range (dark red). Area with lower deposition than our range are located in the arctic (yellow) and area with higher deposition rate are located in highly anthropized areas (black).

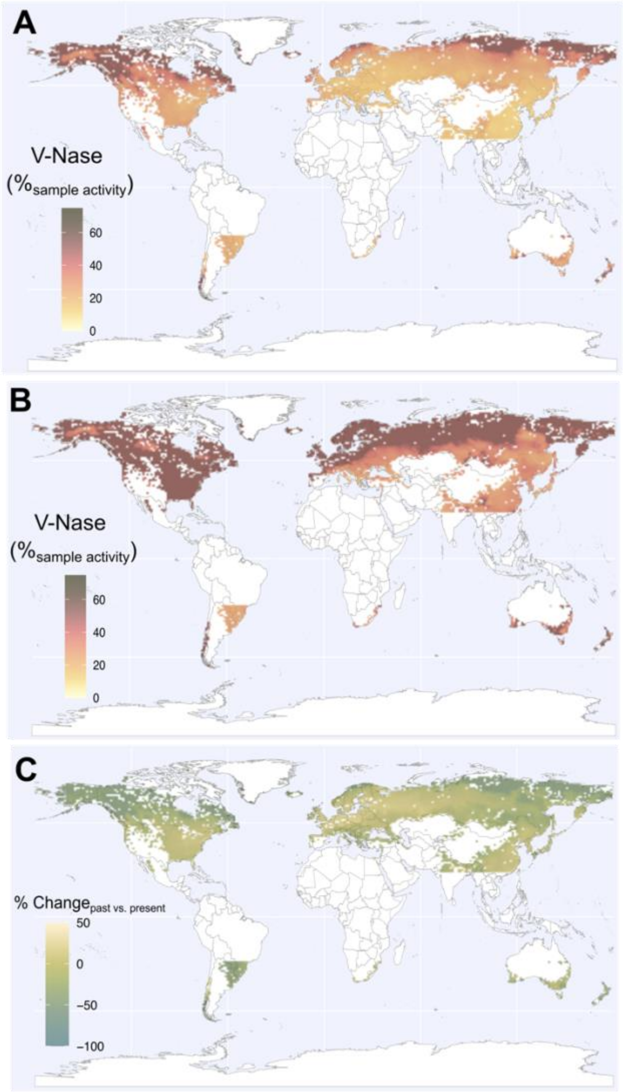

Figure S4: Hotspot of potential V-Nase contribution to N fixation in individual samples of cryptogamic covers in extratropical forests using log_{10} Mo-only model (Rel. 1.2 in SI Table S8). (A) Current and (B) pre-industrial estimates of V-Nase contribution based on deposition of Mo [16,18]. Panel (C) represents the relative change (in % of change) between (A) and (B).

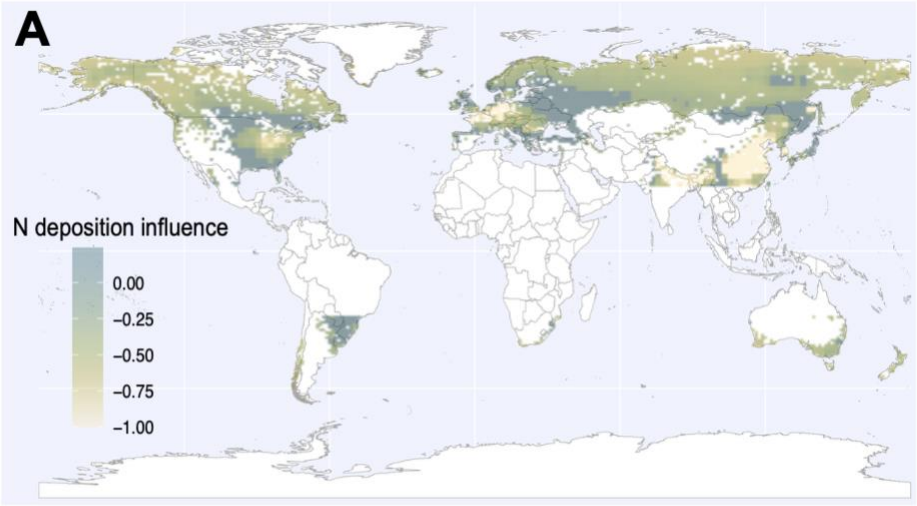

Figure S5: Partial effect of including N deposition (i.e., BNF from Rel. 3) on the potential V-Nase contribution to N fixation in individual samples of cryptogamic covers in extratropical forests. Including N deposition generally further decreases the contribution of V-Nase in urbanized areas (large negative effect in yellow) and in high latitude places to a lower extend (small to moderate negative effect in green). It increases slightly the contribution in other temperate areas (small positive effect in blue). Partial effect of BNF was calculated as $\log_{10}$ of the first order derivative, with negative value indicating a decrease in estimate when incorporating BNF in the model.

A

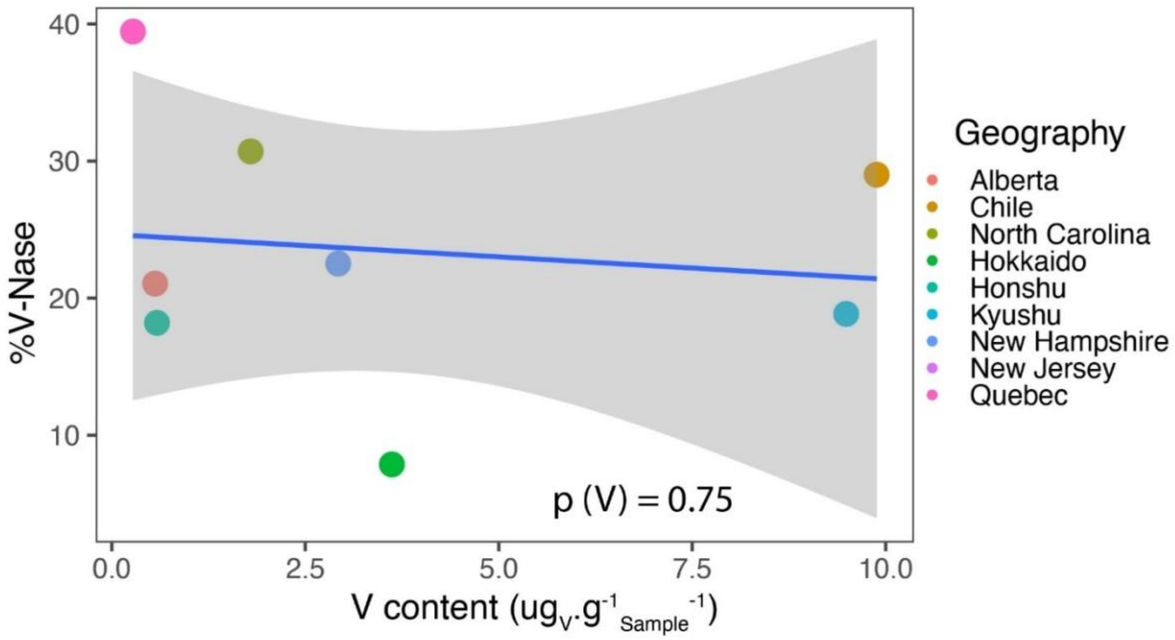

B

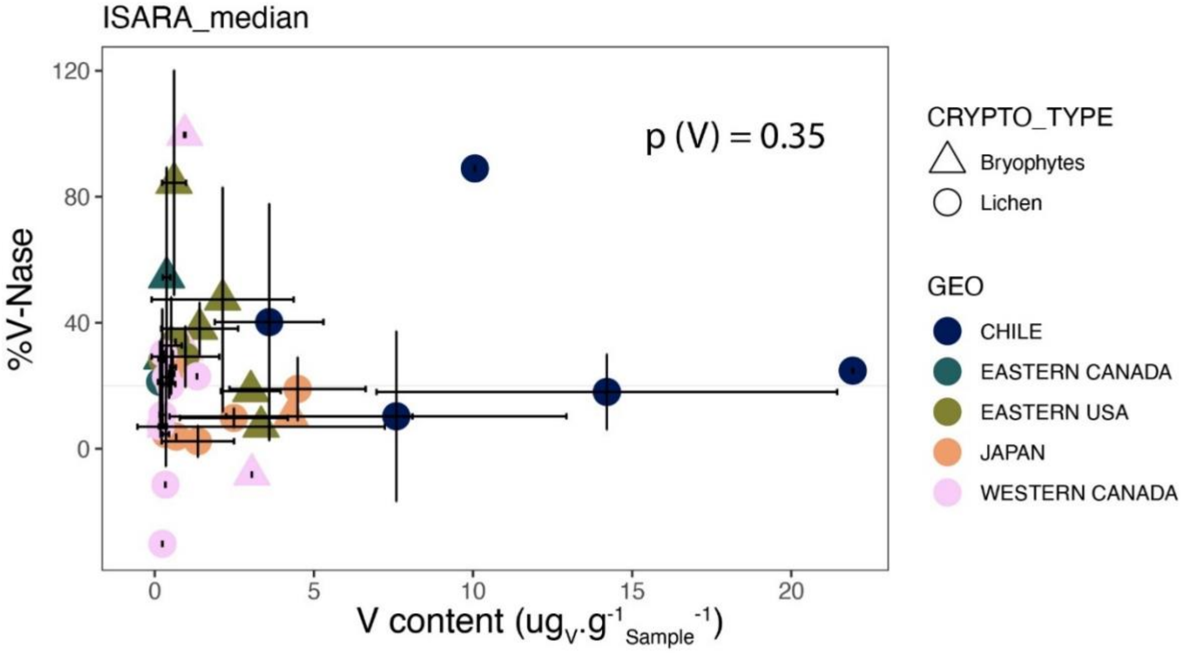

Figure S6: Absence of influence of V metal concentration in sample on V-Nase activity at the location level (n=9) (A), and at the site level (n=38). Grey band represent the 95% prediction intervals. The solid blue line shows the regression line.

Contributor Roles Taxonomy (CRediT):

(Conceptualization, Data curation, Formal Analyses, Funding Acquisition, Investigation, Methodology, Project administration, Resource, Software, Supervision, Validation, Visualization, Writing -original draft, Writing – Review & editing)

Darnajoux Romain[1,2] : Conceptualization, Data curation, Formal analyses, Funding acquisition, Investigation, Methodology, Project administration, Validation, Visualization, Writing - original draft

Shannon Haynes[1]: Investigation, Data curation, Validation, Writing – Review & editing

Marie Renaudin[3#]: Investigation, Writing – Review & editing

Nicolas Magain[4] : Investigation, Resources, Writing – Review & editing

Jolanta Miadlikowska[5] : Investigation, Writing – Review & editing

Diane Haughland[7,8] : Resources, Writing – Review and editing

Yoshitaka Uchida[6] : Resources, Writing – Review and editing

François Lutzoni[5] : Conceptualization, Funding acquisition, Writing – Review and editing

Jean-Philippe Bellenger[3] : Resource, Conceptualization, Writing – Review and editing

Xinning Zhang[1,2] : Conceptualization, Funding Acquisition, Project administration, Resource, Supervision, Writing – Review and editing.

SUPPLEMENTARY LITTERATURE:

(1) Darnajoux, R.; Lutzoni, F.; Miadlikowska, J.; Bellenger, J.-P. Determination of Elemental Baseline Using Peltigeralean Lichens from Northeastern Canada (Québec): Initial Data Collection for Long Term Monitoring of the Impact of Global Climate Change on Boreal and Subarctic Areas in Canada. *Science of The Total Environment* **2015**, *533*, 1–7. https://doi.org/10.1016/j.scitotenv.2015.06.030.

(2) Galloway, J. N.; Dentener, F. J.; Capone, D. G.; Boyer, E. W.; Howarth, R. W.; Seitzinger, S. P.; Asner, G. P.; Cleveland, C. C.; Green, P. A.; Holland, E. A.; Karl, D. M.; Michaels, A. F.; Porter, J. H.; Townsend, A. R.; Vo, C. J. Nitrogen Cycles : Past , Present , and Future. *Biogeochemistry* **2004**, *70*, 153–226.

(3) Darnajoux, R.; Magain, N.; Renaudin, M.; Lutzoni, F.; Bellenger, J. P.; Zhang, X. Molybdenum Threshold for Ecosystem Scale Alternative Vanadium Nitrogenase Activity in Boreal Forests. *Proc Natl Acad Sci U S A* **2019**, *116* (49), 24682–24688. https://doi.org/10.1073/PNAS.1913314116/SUPPL_FILE/PNAS.1913314116.SD01.XLSX.

(4) Vitousek, P. M. The Regulation of Element Concentrations in Mountain Streams in the Northeastern United States. *Ecol Monogr* **1977**, *47* (1), 65–87. https://doi.org/10.2307/1942224.

(5) Goodale, C. L.; Aber, J. D.; Vitousek, P. M. An Unexpected Nitrate Decline in New Hampshire Streams. *Ecosystems* **2003**, *6* (1), 75–86. https://doi.org/10.1007/S10021-002-0219-0/METRICS.

(6) Vitousek, P. M.; Reiners, W. A. Ecosystem Succession and Nutrient Retention: A Hypothesis. *Bioscience* **1975**, *25* (6), 376–381. https://doi.org/10.2307/1297148.

(7) Horn, H. S. Forest Succession. *Sci Am* **1975**, *232* (5), 90–98. https://doi.org/10.1038/SCIENTIFICAMERICAN0575-90.

(8) Haynes, S. J.; Darnajoux, R.; Han, E.; Oleynik, S.; Zimble, E.; Zhang, X. Quantification of Biological Nitrogen Fixation by Mo-Independent Complementary Nitrogenases in Environmental Samples with Low Nitrogen Fixation Activity. *Scientific Reports 2022 12:1* **2022**, *12* (1), 1–12. https://doi.org/10.1038/s41598-022-24860-9.

(9) Zhang, X.; McRose, D. L.; Darnajoux, R.; Bellenger, J.-P.; Morel, F. M. M. M.; Kraepiel, A. M. L. L. Alternative Nitrogenase Activity in the Environment and Nitrogen Cycle Implications. *Biogeochemistry* **2016**, *127* (2–3), 189–198. https://doi.org/10.1007/s10533-016-0188-6.

(10) Bellenger, J.-P.; Xu, Y.; Zhang, X.; Morel, F. M. M.; Kraepiel, A. M. L. Possible Contribution of Alternative Nitrogenases to Nitrogen Fixation by Asymbiotic N_2-Fixing Bacteria in Soils. *Soil Biol Biochem* **2014**, *69*, 413–420. https://doi.org/10.1016/j.soilbio.2013.11.015.

(11) Villarreal, A. J. C.; Renaudin, M.; Beaulieu-Laliberté, A.; Bellenger, J.-P. Stigonema Associated with Boreal Stereocaulon Possesses the Alternative Vanadium Nitrogenase. *Lichenologist* **2021**, *53* (2), 215–220. https://doi.org/10.1017/S0024282921000062.

(12) Nübel, U.; Garcia-Pichel, F.; Muyzer, G. PCR Primers to Amplify 16S RRNA Genes from Cyanobacteria. *Appl Environ Microbiol* **1997**, *63* (8), 3327–3332. https://doi.org/10.1128/AEM.63.8.3327-3332.1997.

(13) Schmittgen, T. D.; Livak, K. J. Analyzing Real-Time PCR Data by the Comparative CT Method. *Nature Protocols 2008 3:6* **2008**, *3* (6), 1101–1108. https://doi.org/10.1038/nprot.2008.73.
(14) Pearson, K. Mathematical Contributions to the Theory of Evolution.—On a Form of Spurious Correlation Which May Arise When Indices Are Used in the Measurement of Organs. *Proceedings of the Royal Society of London* **1897**, *60* (359–367), 489–498. https://doi.org/10.1098/RSPL.1896.0076.
(15) Sutherland, C.; Hare, D.; Johnson, P. J.; Linden, D. W.; Montgomery, R. A.; Droge, E. Practical Advice on Variable Selection and Reporting Using Akaike Information Criterion. *Proceedings of the Royal Society B: Biological Sciences* **2023**, *290* (2007). https://doi.org/10.1098/RSPB.2023.1261/ASSET/FA4B57C0-6279-490D-8878-A1430B940A32/ASSETS/IMAGES/LARGE/RSPB20231261F06.JPG.
(16) Wong, M. Y.; Rathod, S. D.; Marino, R.; Li, L.; Howarth, R. W.; Alastuey, A.; Alaimo, M. G.; Barraza, F.; Carneiro, M. C.; Chellam, S.; Chen, Y.; Cohen, D. D.; Connelly, D.; Dongarra, G.; Gómez, D.; Hand, J.; Harrison, R. M.; Hopke, P. K.; Hueglin, C.; Kuang, Y.; Lambert, F.; Liang, J.; Losno, R.; Maenhaut, W.; Milando, C.; Monteiro, M. I. C.; Morera-Gómez, Y.; Querol, X.; Rodríguez, S.; Smichowski, P.; Varrica, D.; Xiao, Y.; Xu, Y.; Mahowald, N. M. Anthropogenic Perturbations to the Atmospheric Molybdenum Cycle. *Global Biogeochem Cycles* **2021**, *35* (2), 12. https://doi.org/10.1029/2020GB006787.
(17) Tian, H.; Yang, J.; Lu, C.; Xu, R.; Canadell, J. G.; Jackson, R. B.; Arneth, A.; Chang, J.; Chen, G.; Ciais, P.; Gerber, S.; Ito, A.; Huang, Y.; Joos, F.; Lienert, S.; Messina, P.; Olin, S.; Pan, S.; Peng, C.; Saikawa, E.; Thompson, R. L.; Vuichard, N.; Winiwarter, W.; Zaehle, S.; Zhang, B.; Zhang, K.; Zhu, Q. The Global N2O Model Intercomparison Project. *Bull Am Meteorol Soc* **2018**, *99* (6), 1231–1251. https://doi.org/10.1175/BAMS-D-17-0212.1.
(18) Wong, M. Y.; Mahowald, N. M.; Marino, R.; Williams, E. R.; Chellam, S.; Howarth, R. W. Natural Atmospheric Deposition of Molybdenum: A Global Model and Implications for Tropical Forests. *Biogeochemistry* **2020**, *149* (2), 159–174. https://doi.org/10.1007/s10533-020-00671-w.
